# Placozoan fiber cells: mediators of innate immunity and participants in wound healing

**DOI:** 10.1038/s41598-021-02735-9

**Published:** 2021-12-02

**Authors:** Tatiana D. Mayorova, Katherine Hammar, Jae H. Jung, Maria A. Aronova, Guofeng Zhang, Christine A. Winters, Thomas S. Reese, Carolyn L. Smith

**Affiliations:** 1 Laboratory of Neurobiology, National Institute of Neurological Disorders and Stroke, National Institutes of Health, 49 Convent Drive, Bethesda, MD 20892 USA; 2 Central Microscopy Facility, Marine Biological Laboratory, 7 MBL Street, Woods Hole, MA 02543 USA; 3 Laboratory of Cellular Imaging and Macromolecular Biophysics, National Institute of Biomedical Imaging and Bioengineering, National Institutes of Health, 9000 Rockville Pike, Bethesda, MD USA; 4 Light Imaging Facility, National Institute of Neurological Disorders and Stroke, National Institutes of Health, 35 Convent Drive, Bethesda, MD 20892 USA

**Keywords:** Cell biology, Evolution, Zoology

## Abstract

Placozoa is a phylum of non-bilaterian marine animals. These small, flat organisms adhere to the substrate via their densely ciliated ventral epithelium, which mediates mucociliary locomotion and nutrient uptake. They have only six morphological cell types, including one, fiber cells, for which functional data is lacking. Fiber cells are non-epithelial cells with multiple processes. We used electron and light microscopic approaches to unravel the roles of fiber cells in *Trichoplax adhaerens*, a representative member of the phylum. Three-dimensional reconstructions of serial sections of *Trichoplax* showed that each fiber cell is in contact with several other cells. Examination of fiber cells in thin sections and observations of live dissociated fiber cells demonstrated that they phagocytose cell debris and bacteria. In situ hybridization confirmed that fiber cells express genes involved in phagocytic activity. Fiber cells also are involved in wound healing as evidenced from microsurgery experiments. Based on these observations we conclude that fiber cells are multi-purpose macrophage-like cells. Macrophage-like cells have been described in Porifera, Ctenophora, and Cnidaria and are widespread among Bilateria, but our study is the first to show that Placozoa possesses this cell type. The phylogenetic distribution of macrophage-like cells suggests that they appeared early in metazoan evolution.

## Introduction

Members of the phylum Placozoa are flat ciliated marine animals that adhere to and locomote on the substrate. Placozoa are of interest in the context of understanding metazoan evolution because they are widely thought to be sister to the clade that includes Cnidaria and Bilateria^1–8^, although some researchers unite Placozoa and Cnidaria into a clade that is sister to Bilateria^9^. Molecular clock studies indicate that Placozoa diverged > 700 million years ago^10^. Porifera and Ctenophora are thought to have originated earlier, although which evolved first is currently debated^1,5,8,11^.

Nearly two dozen genetically distinct lineages of placozoans have been identified^12^. A clone of *Trichoplax adhaerens* established by the German zoologist Karl Grell in 1970 has been the focus of much research on Placozoa.

*Trichoplax* are typically less than a few millimeters in diameter and 25 µm thick^13–15^. Their bodies are disk-shaped and frequently change in outline due to their amoeboid-like movements^12–14,16^. They adhere to and crawl on surfaces propelled by cilia protruding from cells in their lower epithelium, which exert traction on the substrate as they beat^17^. They feed on algae and cyanobacteria, which they find by chemotaxis and then digest and ingest with their gut-like ventral (lower) epithelium^14,17–20^.

*Trichoplax* have only six broadly defined cell types all but two of which are incorporated into their epithelium^15^. The most prevalent cells in the ventral epithelium are the monociliated ventral epithelial cells (VEC) that propel crawling^15,17,21^. Interspersed among the VEC are lipophil cells that secrete enzymes to digest algae during feeding^17,20^. Semi-digested food is taken up by the VEC through endocytosis^18,21^. The ventral epithelium also contains mucocytes and several types of peptidergic gland cells^22–24^. The dorsal epithelium is much thinner than the ventral epithelium. It is composed of polygonal disks that are extensions of cells whose cell bodies lie below the epithelium^14,15^. Dorsal epithelial cells (DEC) undergo cycles of ultrafast contraction and expansion, which can be sporadic single-cell events or propagating waves^25^. All epithelial cells are joined by adherens junctions through which molecules < 10 kDa freely diffuse. Neither tight, nor septate nor gap junctions nor a basal lamina are present^18^.

Only two cell types reside in the interior of the animal: crystal cells, which are reported to be functional statocysts and are located in a zone near the rim of the animal^15,26^; and fiber cells, which are evenly distributed in a thin layer between the dorsal and ventral epithelium^15,27^. Fiber cells have long branching processes that contact other fiber cells as well as cells in the dorsal and ventral epithelia^15,28^. Their processes form the only internal link between the dorsal and ventral epithelial cells, thereby fulfilling an important structural role similar to mesogleal cells in Cnidaria and mesenchymal cells in Bilateria. However, in contrast to mesoglea and mesenchyme, the fiber cell layer in *Trichoplax* contains very little extracellular matrix^15,28,29^.

Contacts between fiber cells and adjacent cells typically are punctate and have only sparse material in the intercellular cleft^15,24,26^. Some fiber cells are interconnected by syncytial “plug” junctions^15,30^ resembling those between electrically coupled cells of sponges^31,32^ and colonial choanoflagellates^33,34^. However, such junctions are very rare and therefore seem unlikely to support communication among the entire population of fiber cells^30^.

One of the distinguishing morphological features of fiber cells is their content of one or more large, phagosome-like inclusions. Food particles (remnants of algae or yeast) have been observed in fiber cell phagosomes^35,36^, leading to speculation that fiber cells might participate in feeding by a process called transepithelial cytophagy.

Several authors have attributed muscle-like contractility to fiber cells^27,37,38^, although no experimental support for this idea has been documented. The processes of dissociated fiber cells are reported to twitch and to extend and retract^38^, behaviors that are common to many motile cell types.

An RNAseq study of cell types in *Trichoplax* identified a cell cluster that was postulated to represent fiber cells based on the expression of genes associated with cell motility and extracellular matrix^39^. Cells in this cluster also expressed the transcription factor *forkhead box C* (*foxC)*^39^, which is expressed in the somatic gonad, a nutritive structure, in Cnidaria^40^ and in mesoderm-derived cells in Bilateria^41–43^.

To date, motile mesenchymal cells have been described in Ctenophora, Porifera, Cnidaria and Bilateria, where their role is largely attributed to immunity, structural organization of tissues, proliferation, and wound healing (reviewed by^44–46^). As fiber cells reside in the interior of placozoans, interact with other cell types and are capable of phagocytosis, we supposed that they might perform functions associated with macrophage-like cells in other animals.

The present study was undertaken to gain a more complete picture of the structure and function of fiber cells and to discover marker genes that can be used to identify them. We find that fiber cells are motile phagocytes that engulf cell debris and bacteria and also participate in wound healing. They express *foxC* as well as genes implicated in phagocytosis and cell motility, as was inferred in the scRNAseq study^39^. These findings complement and extend previous studies documenting the similarities in morphology, patterns of gene expression and function between cell types in Placozoa and other metazoans.

## Results

### Fiber cells and their milieu.

**Figure 1:**
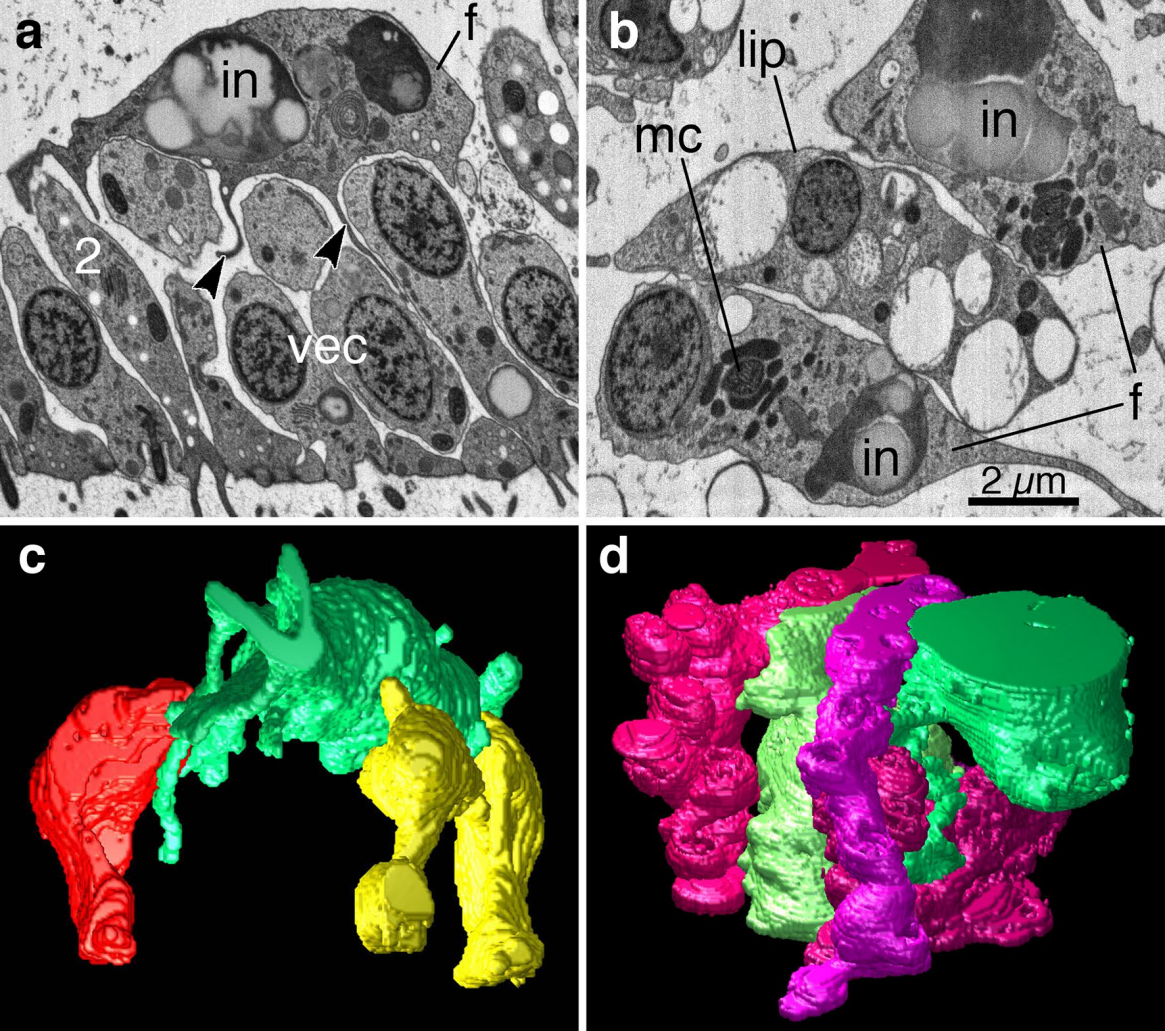
Fiber cell morphology and contacts with other cell types. (**a**) Thin section through a fiber cell (f) in close apposition to the basal parts of ventral epithelial cells (vec) and Type 2 gland cell (2). Processes of the fiber cell (arrowheads) extend into the clefts between adjacent epithelial cells. (**b**) Thin section through two fiber cell bodies flanking a lipophil cell body (lip) in the interior of the animal.(**c**, **d**) Surface renderings generated from 123 and 111 sections, respectively, illustrate fiber cells (green) in close proximity to other cell types: VEC (yellow) and Type 2 gland cell (red) in a marginal zone (**c**) and lipophils (different shades of magenta) in a lipophil zone (**d**). For clarity, only a subset of the cells contacted by the fiber cells was included in the reconstruction. Thin sections obtained with an ATLUM were imaged with a SEM in backscatter mode. *in* fiber cell inclusion; *mc* mitochondrial complex.

To obtain a more comprehensive and detailed view of the contacts of fiber cells with other cell types than provided previously^15^ we used scanning electron microscopy (SEM) of serial sections^47^. Fiber cells were easily recognized due to their possession of processes, large inclusions and a mitochondrial complex consisting of mitochondria interspersed with pale inclusions whose identity is unknown^15,30,37^. The *Trichoplax* body manifests two zones^24^: (1) *a marginal zone*, approximately 100 µm wide, starting at the rim where the dorsal and ventral epithelia meet, and (2) *a lipophil zone*, occupying the rest of the animal, and characterized by the presence of lipophil cells, which have a digestive role^17^. Fiber cells were present throughout the lipophil zone and in all but the outer 10 µm of the marginal zone. In the marginal zone, fiber cell bodies were located close to the basal parts of VEC (Fig. 1a,c; Supplementary Movie S1) and the two morphologically distinct types of gland cells (Type 1, not illustrated; Type 2, Fig. 1a,c) that reside in this zone^24^. Fiber cells also contacted crystal cells, located beneath the epithelium near the rim^26^. In the lipophil zone, fiber cell bodies were interspersed with the bodies of lipophil cells (Fig. 1b,d; Supplementary Movie S2), which extended deeper into the interior than did VEC bodies, and gland cells (Types 2 and 3; not illustrated). Fiber cell processes extended ventrally between the cell bodies of the VEC, transversely around the cell bodies of fiber and lipophil cells and dorsally to the DEC. Fiber cell contacts, defined as areas of close apposition with the membrane of another cell showing material in the intercellular cleft, were observed with all cell types although, as noted previously^15,24^ the contact areas were small. Syncytial (plug) junctions^15,30^ were not visible at the sampling resolution we used for SEM.

**Figure 2:**
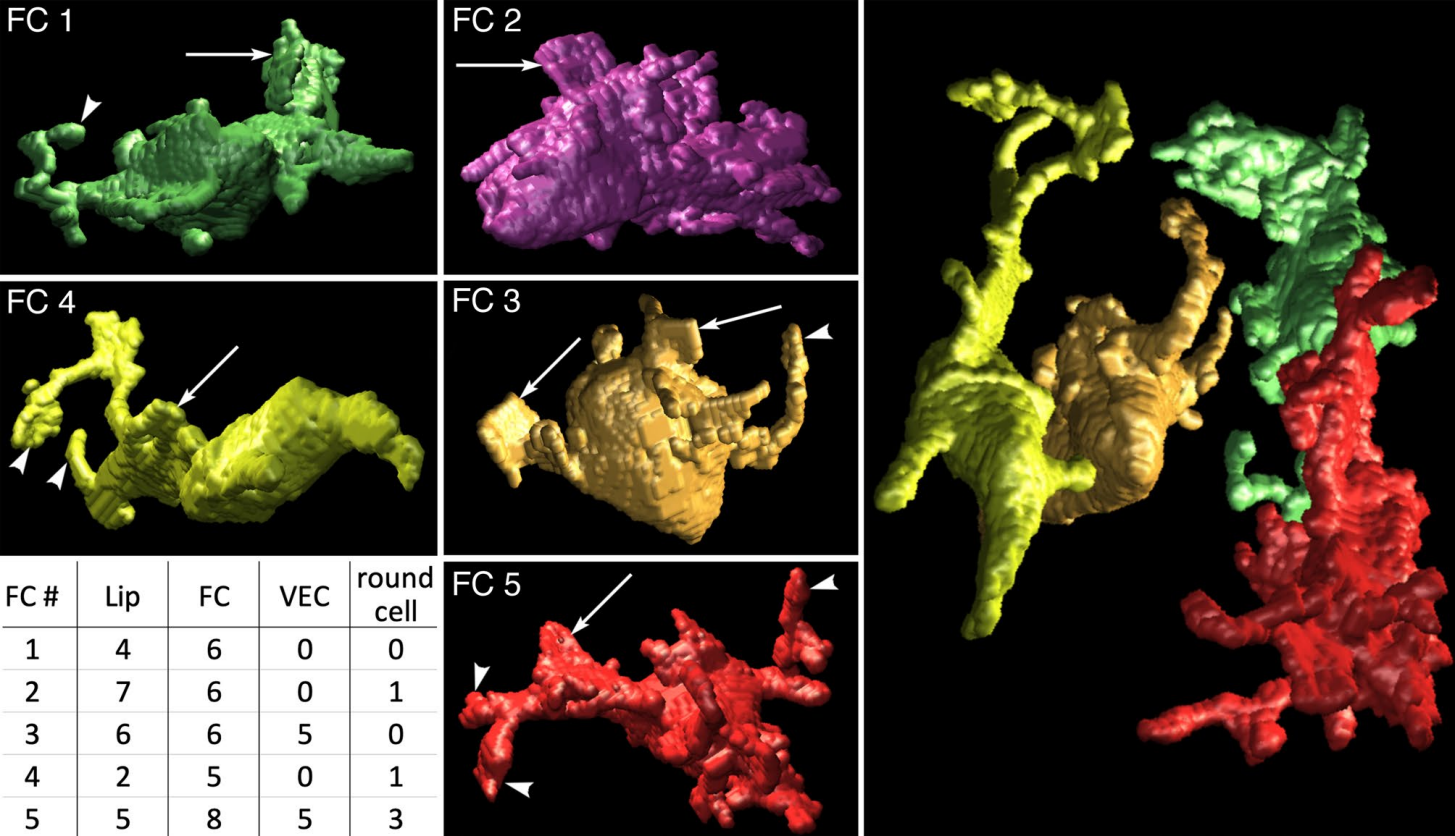
Three dimensional reconstructions of five fiber cells (FC 1–5) located close to each other within the lipophil zone. Surface renderings demonstrate that some fiber cell processes are flat and wide (arrows) while other are round and thin (arrowheads). Wide processes of FC 4 and 5 extend narrower branches that protrude in different directions. Right panel shows arrangement of FC 1 and 3–5. The table shows the number of each cell type contacted by the fiber cells. The data was generated from a 195-section series obtained by SBF-SEM. *Lip* lipophil cell.

We used serial blockface SEM (SBF-SEM) at lower resolution (10 nm x, y; 100 nm z) to obtain a larger contiguous series that completely encompassed five fiber cells in the lipophil zone. Surface renderings of the fiber cells revealed the diverse shapes of their protrusions (Fig. 2, Supplementary Movie S3). Some of their processes were flat and wide whereas others were thin and round in cross section. Many of the processes branched, and wide flat processes often bore thin branches (Fig. 2, Supplementary Movie S3). Examination of the cells in serial sections allowed us to identify and enumerate the cells with which they interacted, as evident by the presence of an extended region of close proximity. Each fiber cell interacted with multiple lipophil cells (4.8 ± 1.9, Mean ± StDev) and fiber cells (6.2 ± 1.1; see table in Fig. 2). No anastomoses between fiber cell processes were detected. Two fiber cells extended processes into the ventral epithelium and each interacted with five VEC (Fig. 2). Three fiber cells also interacted with small, rounded cells. This cell type has not been previously detected in *Trichoplax*. The overall shape of these cells was round with no processes. The only organelles visible at the resolution of SBF-SEM were the nucleus and a small round inclusion typical for VEC. These cells were similar in appearance to cells reported in another placozoan, *Hoilungia hongkongensis*, and suggested to be recently divided^48^.

To gain insight into possible functions of fiber cells, we examined whether fiber cells are distributed randomly or in a pattern. We used immunofluorescence images of five wholemounts labelled with an antibody that marks the surfaces of fiber cells (anti-FC^15^; see “Methods” for a description of the antibody). The distances between the centroids of adjacent fiber cells were measured in maximum intensity projections of optical sections collected by confocal microscopy. The distance between the centroids of fiber cell bodies ranged from 7.44 to 10.34 µm (Supplementary Table S1). These measurements were used to determine whether the distribution of fiber cell centroids is random, clustered, or self-avoiding^49^. Comparison of the average separation between fiber cell centroids consistently detected a self-avoiding pattern that differed significantly from a random distribution (Welch’s t-test, p < 0.05).

### Fiber cells manifest phagocytic activity

#### Phagocytosis of dead cells and cell debris

**Figure 3:**
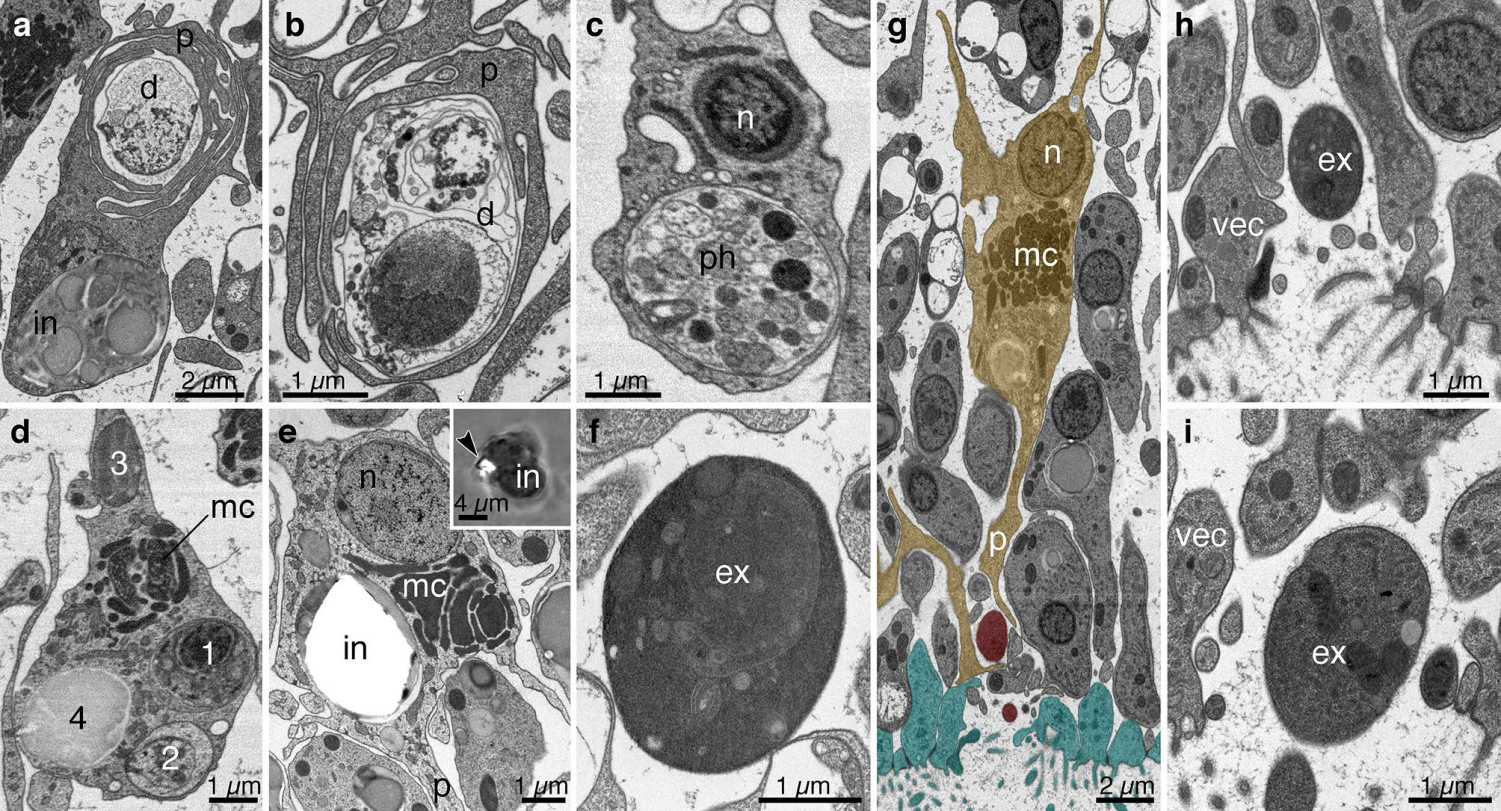
Fiber cell involvement in phagocytosis of unhealthy/dead cells. (**a**) A fiber cell containing a characteristic inclusion (in) encircles cell debris (d) with multiple branching processes (p). (**b**) Fiber cell processes wrapped around a dead cell (d) containing nuclear and vesicular remnants. (**c**) Phagosome (ph) with heterogeneous and vesicular content, likely representing recently phagocytosed cell debris. (**d**) A fiber cell with four phagosomes (1–4) exhibiting different degrees of processing/digestion: phagosomes 1 and 2 still have remnants of cell organelles, while phagosomes 3 and 4 are more homogeneous and may represent later stages of processing. (**e**) A fiber cell with an empty space in its inclusion corresponding in shape and size to a crystal in a crystal cell; inset shows a differential interference contrast (DIC) image of a living fiber cell with a crystal (arrowhead) inside it, perhaps representing a phagocytosed crystal cell. (**f**–**i**) Extracellular residual bodies. (**f**) An extracellular dark particle (ex) in the interior of the animal. The content of the particle is electron dense and relatively homogeneous, similar to phagosomes observed in the interior of fiber cells (**d**, phagosome 3). (**g**) Fiber cell processes (mustard) associated with extracellular dark particles (red) located near gaps between cells in the ventral epithelium (cyan). (**h**) The extracellular dark particle near the mouth of the gap in a different section from the series shown in g. (**i**) A dark particle located outside of another animal near a breach in the ventral epithelium. (**a**, **b**, **e**–**i**)—TEM; (**c**, **d**)—SEM in backscatter mode. *mc* mitochondrial complex, *n* fiber cell nucleus.

A subset of the fiber cells had multiple processes wrapped around cells with pale cytoplasm, swollen organelles and electron dense inclusions (Fig. 3a,b). Since the appearance of the content of enwrapped cells was strikingly different from that of all other surrounding cells, we assume the cells were unhealthy. Other fiber cells had large phagosomes with highly polymorphic, granular and membranous material consisting of multiple vacuoles of different size and electron density (Fig. 3c). Since the content of these phagosomes resembled cell debris, it may well represent recently phagocytosed unhealthy cells. We also observed fiber cell phagosomes with more homogeneous and electron dense content, which likely reflects more advanced stages of degradation of phagocytosed cell debris. Some fiber cells had multiple phagosomes containing cellular debris at different stages of degradation (Fig. 3d) including early-stage phagosomes containing vesicles and remnants of nuclei (Fig. 3d, inclusions 1 and 2) and phagosomes at advanced stages with more homogeneous electron dense content (Fig. 3d, inclusion 3). Phagocytic fiber cells also had an inclusion containing large (typically from 3 to 10 µm but can be up to 15 µm) vacuoles whose content was uniformly intermediate in electron density (Fig. 3a,d, inclusion 4). This type of inclusion, referred to as a concrement vacuole^16^, is present in all fiber cells (see Fig. 1a,b).

Fiber cells also phagocytose crystal cells as is evident because some fiber cells contained voids inside their inclusions that were similar in silhouette to the crystal in crystal cells (Fig. 3e). Additional evidence that fiber cells can phagocytose crystal cells came from visualizing living fiber cells by DIC microscopy. Fiber cells, identified by their possession of processes and a large, dark inclusion sometimes had a birefringent crystal inside their inclusion that was similar in size and shape to the crystal in crystal cells (Fig. 3e, inset).

Electron dense particles that were similar in size and appearance to fiber cell inclusions at advanced stages of cell degradation (residual bodies) occurred in the extracellular space of the animal (compare the inclusion 3 in Fig. 3d and the particle in Fig. 3f). These extracellular dark particles were surrounded by a membrane. They were most common in the ventral epithelium, where they were adjacent to fiber cell processes and to VEC bodies (Fig. 3g–i). In an area where a dark particle was close to the ventral surface, the integrity of the ventral epithelium was broken: VEC lost contact with their neighbors, leaving a gap between cells several micrometers in width (Fig. 3g–i). The dark particle was near the mouth of the gap (Fig. 3g,h). In a series of sections from another animal, a dark particle was located outside of the animal near a smaller gap between adjacent VEC (Fig. 3i).

Time lapse imaging of living dissociated cells with DIC and fluorescence optics provided further evidence of the phagocytic behavior of fiber cells. Dissociated fiber cells, identified by their possession of a large and opaque inclusion, adhered to the cover glass and spread a thin lamellum around their circumference (Supplementary Fig. S1 and Movie S4). The fiber cells had multiple processes, some of which waved back and forth, extended, or retracted. Fiber cells plated directly on the coverslip did not migrate, but fiber cells plated on glass coated with Matrigel did migrate (Supplementary Fig. S2). Fiber cells interacted with nearby cells in a manner indicative of phagocytic activity. For example (Supplementary Fig. S1; Supplementary Movie S4), processes of a fiber cell palpated a neighboring cell, likely a VEC in view of its possession of a small opaque inclusion. The VEC may have been unhealthy because its cell body was rounded rather than elongated in outline, as is typical of healthy VEC with a motile cilium. After a period of contacting, two processes of the fiber cell embraced the VEC from opposite sides and eventually fused their tips (Supplementary Fig. S1; Supplementary Movie S4). The VEC was then pulled inwards, and the fiber cell body resumed its rounded/oval shape. The movie captured a thin (800 nm) optical section relative to the diameter of the VEC (~ 5 µm) and fiber cell (~ 8 µm), so we cannot be certain that the fiber cell completely surrounded the VEC. However, the fact that the VEC remained inside the fiber cell for the duration of the movie and exhibited movements like those of organelles within the fiber cell led us to believe that it was phagocytosed.

#### Phagocytosis of bacterial pathogens

**Figure 4:**
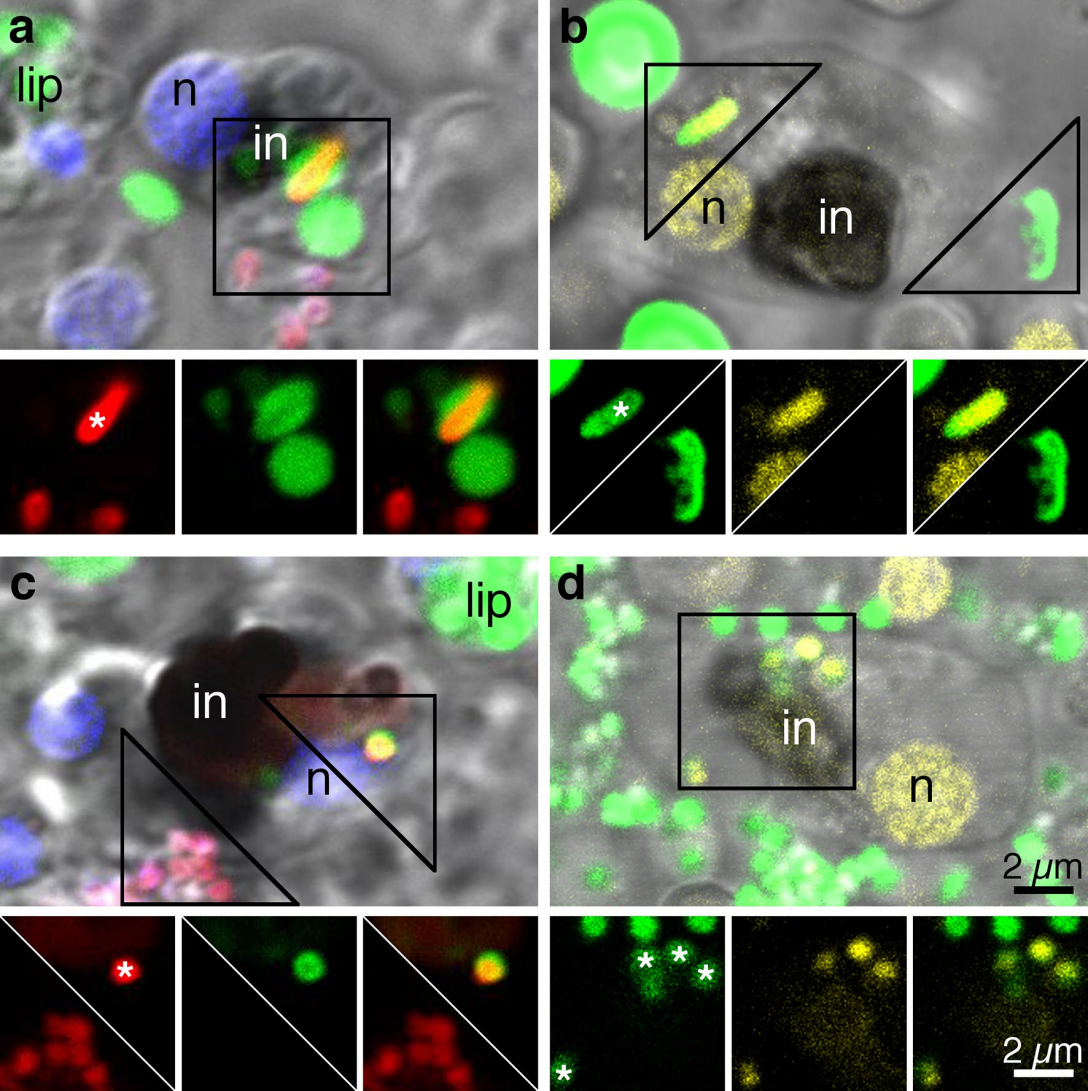
Uptake of bacteria by dissociated fiber cells. Merged DIC and fluorescence images. *Trichoplax* cells challenged with fluorescent Gram negative (**a**, **b**) or Gram positive (**c**, **d**) bacterial bioparticles. (**a**, **c**) Bacteria of both types (red) appear inside round 1–2 µm organelles labelled with lysotracker (green) in fiber cells. (**b**, **d)** Bacteria of both types (green) located inside fiber cells demonstrate DHE labelling, indicating oxidation by ROS in the cell interior. Insets, below, correspond to the framed regions and show fluorescent channels separately and merged. Note co-localization of bacterial fluorescence and vital dye staining associated with phagocytosed bioparticles (asterisk) of both types and the absence of vital dye staining in bioparticles (no asterisk) located outside of fiber cells. Blue labelling in (**a**) and (**c**) is Hoechst nuclear stain. *in* fiber cell inclusion; *lip* lipophil granules labelled with Lysotracker; *n* fiber cell nucleus.

We also examined the interactions of fiber cells with heat killed fluorescent bacteria. Fiber cells engulfed both Gram-negative (*E. coli*, Fig. 4a,b) and Gram-positive (*S. aureus*, Fig. 4c,d) bacteria. We used vital dyes that stain components of the phagocytic pathway to verify that the bacteria were inside the fiber cells. Some bacteria located within the contours of fiber cell were labeled by lysotracker (Fig. 4a,c), an acidophilic dye that stains lysosomes and other acidic organelles, indicating that these bacteria were in the interiors of fiber cells. In another experiment, bacteria located within fiber cell contours manifested labelling by 2-hydroxy-ethidium generated in situ after oxidation of dihydroethidium (DHE) added to ASW (Fig. 4b,d), a marker for reactive oxygen species (ROS). 2-hydroxy-ethidium, a fluorescent molecule that has an affinity to DNA, labelled DNA in the bacteria and in fiber cell nuclei (Fig. 4b,d). By contrast, bacteria that adhered to the surfaces of cells (Fig. 4) or to the cover glass (not illustrated) showed no labeling with lysotracker (Fig. 4a,c) or DHE (Fig. 4b,d). None of the other recognizable and prevalent cell types (lipophil cells, epithelial cells, and crystal cells) in the cultures had bacteria with vital dye labelling within their contours.

### Fiber cells express genes implicated in phagocytosis and innate immunity

**Figure 5:**
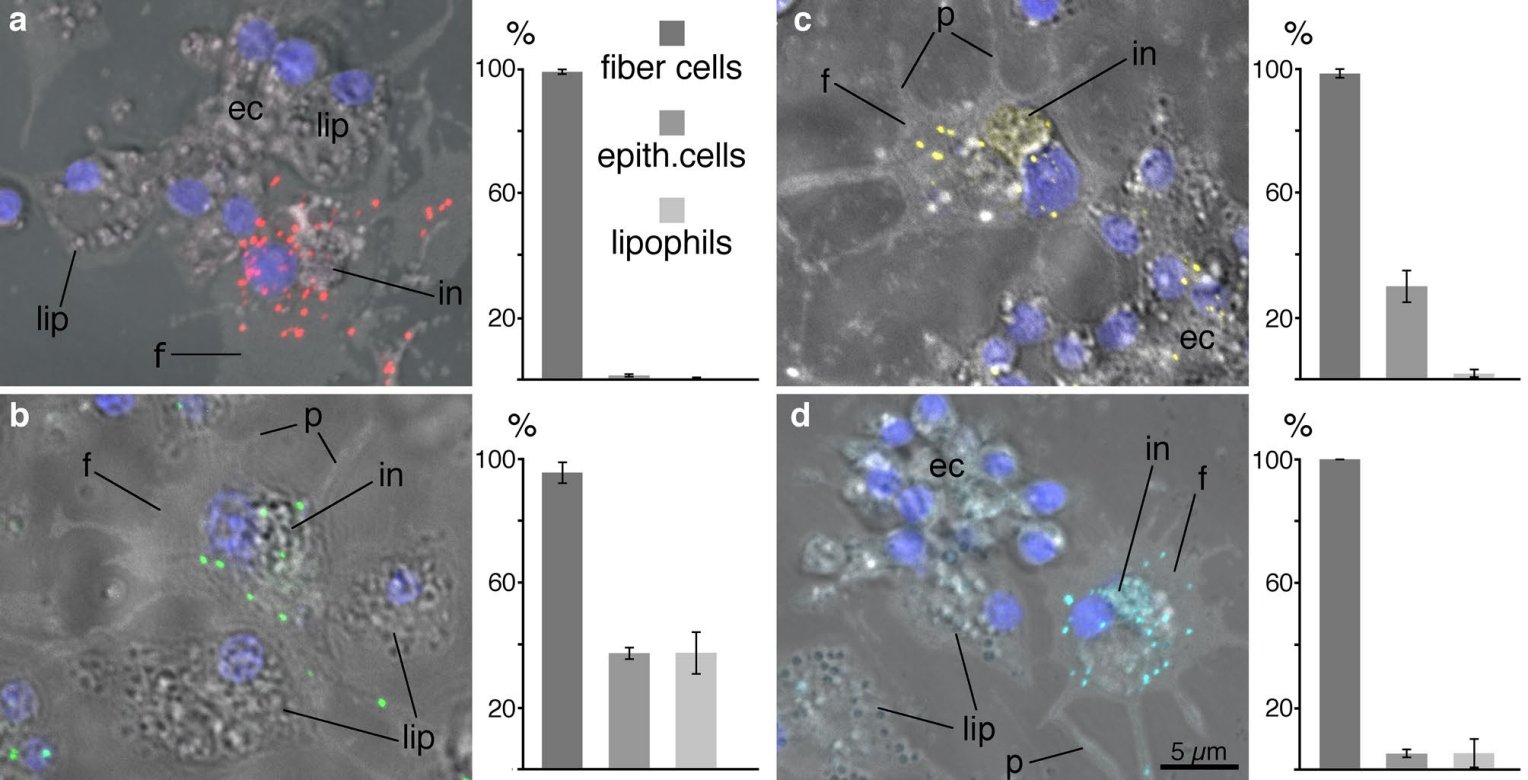
In situ hybridization with probes for the transcription factor *foxC* and for genes implicated in phagocytosis in dissociated cell samples. Images are maximum projections of fluorescence images captured with a confocal microscope superimposed on a transmitted light image. Fiber cells (f) were identified by their possession of a large inclusion (in) and processes (p); lipophil (lip) cells, by their content of numerous refractile vesicles; and spherical/columnar epithelial cells (ec, epithelial cells excluding lipophils and mucocytes), by their small sizes and smaller granules. The graphs show the percent of each cell type that was labeled (mean ± SEM). (**a**) A fiber cell labeled with Ta-*foxC* probe (red) adjacent to unlabeled lipophil and spherical/columnar epithelial cells. *foxC* is highly expressed exclusively in fiber cells. (**b**) A fiber cell labeled with Ta-*cd36* probe (green) flanked by two unlabeled lipophil cells. *cd36* is expressed in almost all fiber cells and in less than half spherical/columnar epithelial cells and lipophil cells. (**c**) A fiber cell labeled with Ta-*elmo* probe (yellow) adjacent to a cluster of labeled and unlabeled spherical/columnar epithelial cells. *elmo* is expressed in almost all fiber cells and in a third of spherical/columnar epithelial cells, but not in lipophil cells. (**d**) A fiber cell labeled with Ta-*pxmp2* probe adjacent to an unlabeled lipophil cell and cluster of spherical/columnar epithelial cells. Expression of *pxmp2* is largely confined to fiber cells.

To study molecular signatures of fiber cells, we first of all needed to identify molecular markers that had strong expression in fiber cells and no or very little expression in other cell types. Data from a single cell RNA sequencing study of *Trichoplax*^39^ suggested likely candidates are the transcription factor *foxC* and *peroxisomal membrane protein 2* (*pxmp2*). We performed in situ hybridization with a fluorescent probe for *foxC* in dissociated cells of *Trichoplax*, where fiber cells, lipophil cells, and clusters of spherical/columnar epithelial cells (likely including DEC, VEC, and some gland cells, but excluding WGA-stained mucocytes) could readily be identified by their morphology. All sampled fiber cells (99.2 ± 4.0%, mean ± StDev) had strong expression of *foxC*; apart from this, a very small portion of spherical/columnar epithelial cells (0.9 ± 1.7%) demonstrated moderate expression (Fig. 5a). These results validated use of *foxC* expression as a specific marker for fiber cells.

We performed dual-color in situ hybridization in whole mounts with probes for *foxC* and genes implicated in phagocytic activity: *cluster of differentiation 36* (*cd36*), *engulfment and cell motility* (*elmo*) *protein*, and *pxmp2*. The expression signals for *foxC* and each of the other genes clustered together (Supplementary Fig. S3), and many of the clusters were in the vicinity of fiber cell inclusions (Supplementary Fig. S3), which were visible due to their autofluorescence. The association of label clusters with fiber cell inclusions suggests that the label might be expressed in fiber cells along with *foxC*. Moreover, the labelling for each probe occurred throughout the interior of the body but was very sparse in the outermost ~ 10 µm of the marginal zone, consistent with localization of fiber cells. Scarce fluorescent grains of *foxC* label in the outermost rim of the marginal zone could be located in the infrequent epithelial cells that express *foxC* (Fig. 5a) or in fiber cell processes that extend into the epithelium (Figs. 1a, 3g).

Analysis of *cd36, elmo* and *pxmp2* expression in dissociated cells (Fig. 5b–d) showed that each gene was expressed in a large fraction of fiber cells: *cd36* (95.6 ± 13.3%); *elmo* (98.5 ± 3.3%); *pxmp2* (100%). *cd36* was expressed by approximately one third of the lipophil cells and one third of the spherical/columnar epithelial cells. Approximately one third of the spherical/columnar epithelial cells expressed *elmo*, but very few lipophil cells expressed *elmo*. Fewer than ten percent of spherical/columnar epithelial cells and lipophil cells expressed *pxmp2*.

### Fiber cells are involved in wound healing

**Figure 6:**
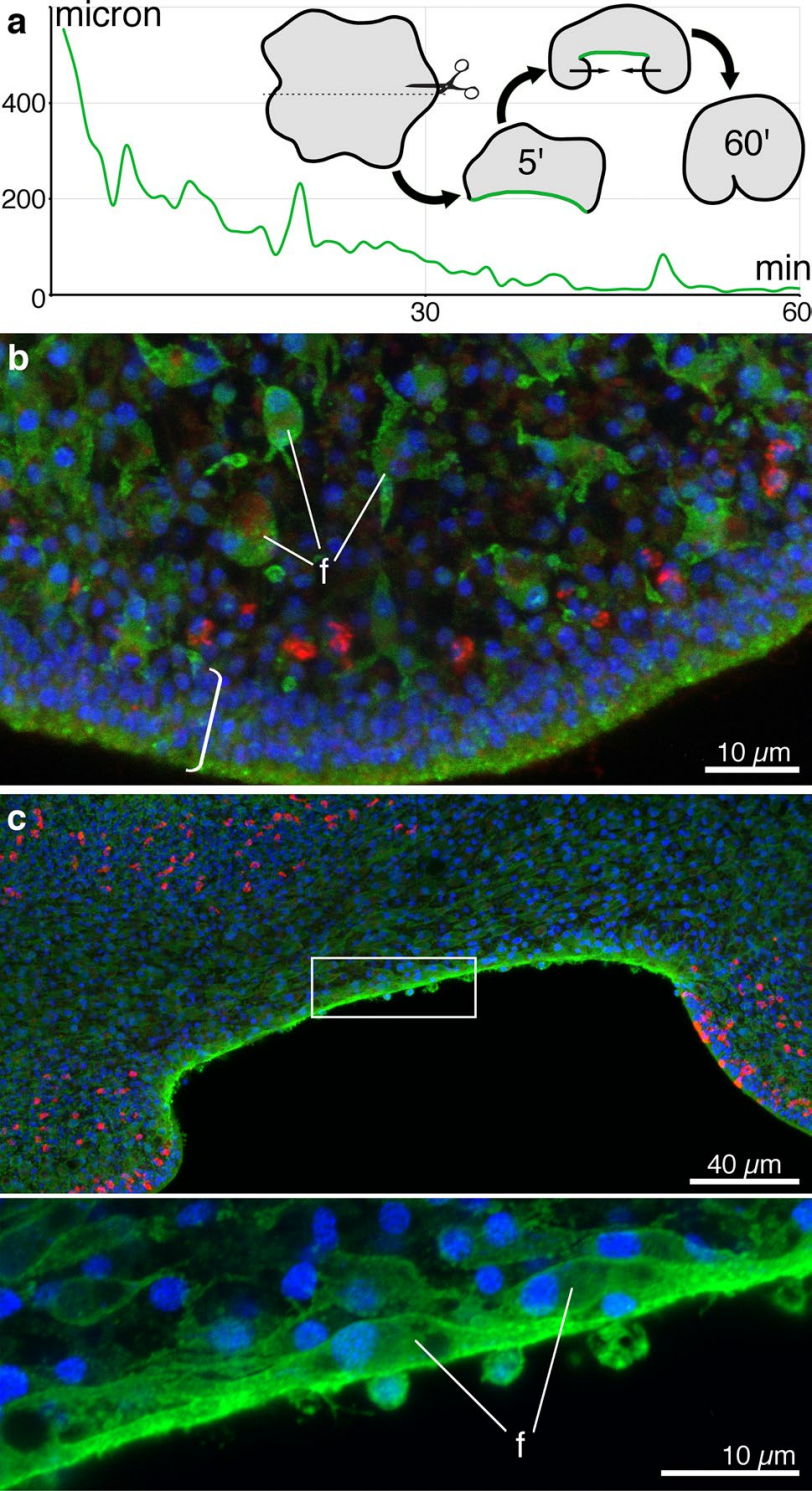
Fiber cell involvement in wound healing in *Trichoplax*. (**a**) Graph illustrating the time course of wound closure in a cut animal (wound length versus time) and a scheme of the experiment and stages of wound healing. Animals were cut in half. Five minutes after cutting, the wounded area (green) was slightly invaginated. The wound margins curled inwards and gradually became closer with intermittent reopenings. The margins met and fused by ~ 60 min, leaving a notch marking the site of the wound. (**b**, **c**) Fluorescence staining for fiber cells (anti-FC, green), mucocytes (WGA, red), and nuclei (Hoechst, blue) in intact and wounded *Trichoplax*. (**b**) In intact *Trichoplax*, tightly packed epithelial cells form a rim (bracket) around the circumference of the body. A row of mucocytes resides within the rim or close inside. Fiber cells (f) are arrayed in the interior starting ~ 10 µm from the edge. (**c**) In wounded *Trichoplax*, fiber cells are aligned along the wound and are more tightly packed and intensely stained than fiber cells in the interior or in intact animals. Inset (framed region) shows magnified view of fiber cells aligned along the wound.

To investigate wound healing, *Trichoplax* were cut in half (Fig. 6a). Time lapse video microscopy of wounded animals showed that the intact epithelia at the two margins of the wound extended toward each other, reducing the area of the wound, and after about one hour fused, thereby closing the wound (Fig. 6a, Supplementary Movie S5). During early stages of this process, the two margins of the wound sometimes moved away from each other, reopening the wound, but as time passed reopenings occurred less frequently.

To determine whether fiber cells participate in wound healing, we stained wholemounts of intact and wounded animals with anti-FC antibodies to label fiber cells, WGA to label mucocytes (Type 2 gland cells), which are prevalent in the marginal zone but also present further in the interior, and Hoechst dye to label nuclei. In intact animals, the outermost 10 µm of the marginal zone was composed of tightly packed VEC and overlaying DEC (Fig. 6b). A row of mucocytes (red cells in Fig. 6b) was located 10 to 30 µm from the rim. Fiber cells were present beginning ~ 10 µm from the edge and extending throughout the marginal zone (Fig. 6b) and lipophil zone (not illustrated). In wounded animals, the cut edge changed strikingly from the intact one. Fiber cell accumulated near the wound edge by five minutes after surgery (Supplementary Fig. S4a). Fiber cells were in contact with the seawater and their cell bodies were stretched along the wound parallel to the cut edge. Thirty minutes after wounding, fiber cell bodies were more elongated and their processes were aligned along the cut edge (Fig. 6c, inset). At both 5 and 30 min, fiber cells exposed directly to the seawater had much brighter anti-FC labelling than fibers cells in the interior. High level of *foxC* expression was apparent along the cut edge, consistent with presence of fiber cells in this area (Supplementary Fig. S4b).

**Figure 7:**
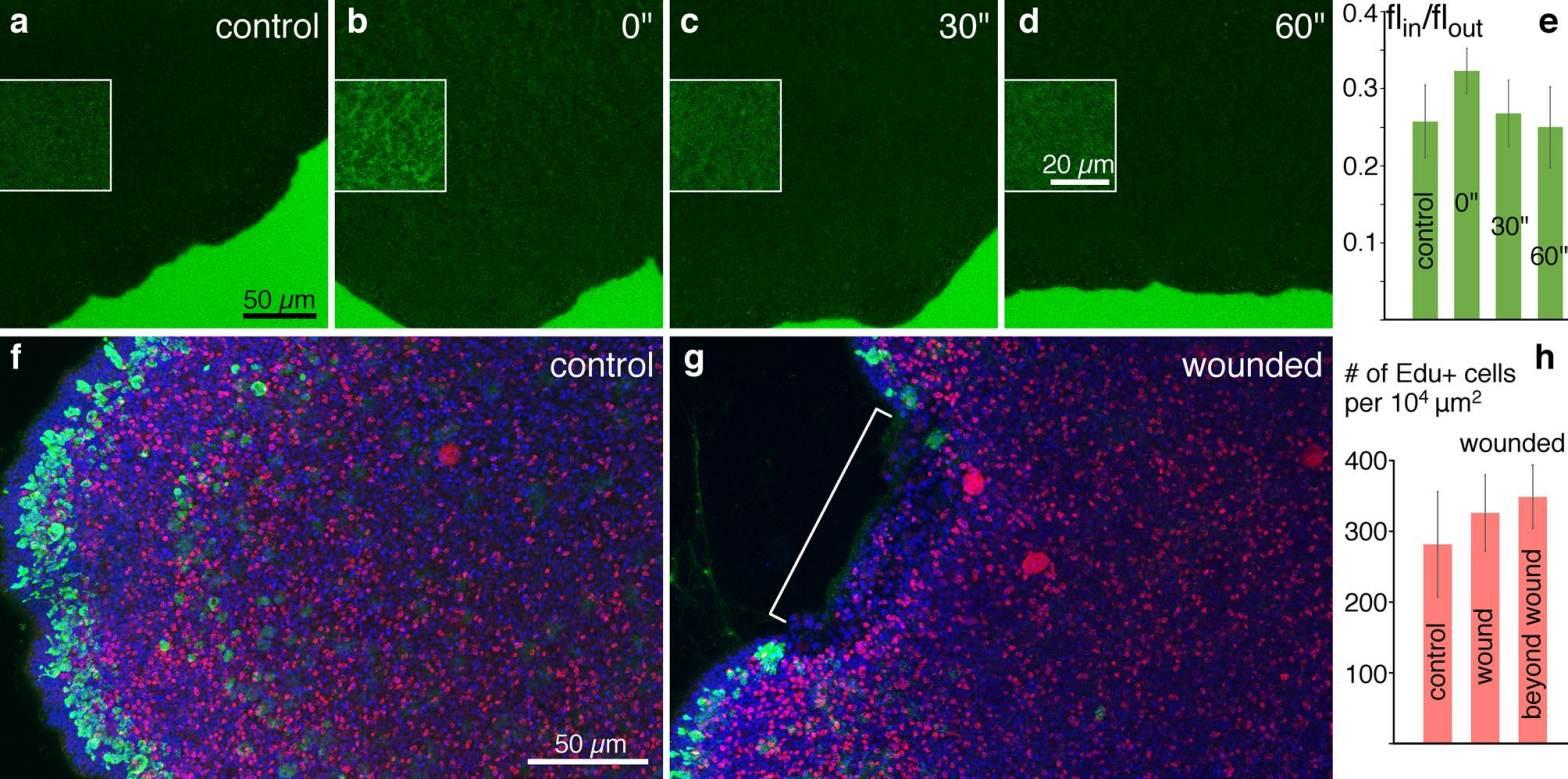
Estimation of the time required to seal the wound and proliferative activity during wound healing. (**a**–**d**) An area encompassing the edge of an animal bathed in 70 kDa FITC conjugated dextran (green); the amount of fluorescence inside the animal reflects the amount of dextran penetrated through the wound. Insets represent an enlarged area with enhanced brightness and contrast. (**a**) Control (intact) animal. (**b**) Dextran added before wounding. (**c**) Dextran added 30 s after wounding. (**d**) Dextran added 60 s after wounding. (**e**) Ratio of inside to outside dextran fluorescence in the animals in each experimental condition; no significant difference was found. Error bars are StDev. (**f, g**) Proliferative activity during 35 min (EdU labelling, red; WGA, green; DAPI, blue) in intact control animal (**f**) and after cutting (**g**, wounded area marked with bracket) 35 min after surgery. (**h**) Mean number of EdU labeled nuclei in 10^4^ µm^2^ in lipophil area. No significant difference was found. Error bars are StDev.

To determine the time course of physical sealing of a wound, we bathed *Trichoplax* in fluorescent 70 kDa dextran, which normally does not penetrate inside the intact animals (Fig. 7a^18^). In animals that were cut in half *after* the dextran was added mesh-like staining was visible inside the body (Fig. 7b). This pattern suggests that extracellular space was stained^18^, as expected if dextran penetrated the wound. In animals that were cut 30 or 60 s *before* dextran application no mesh-like staining was detected (Fig. 7c,d). The amount of dextran entering the animal was very small even when the dextran was added before cutting and the average fluorescence value was not significantly higher than in other groups; no significant difference was apparent by ANOVA (F = 3.236; p = 0.05219; Fig. 7e). While cutting animals exposed fiber cells at the wound edge to seawater (Figs. 6c,d; S4a), it did not create a significant pathway for diffusion of dextran into the interior of the animal.

Mitotic cells visualized by 5-ethynyl-2′-deoxyuridine (EdU) labelling were present in equivalent numbers in wounded and intact animals (Fig. 7f–h). In control animals, EdU positive nuclei were scattered evenly and randomly throughout the body, leaving free only the very edge of the marginal zone (10–15 µm), confined by the mucocyte row (Fig. 7f). The density of EdU positive nuclei did not change in wounded animals: they did manifest a proliferative activity along the wound and in adjacent tissue, but the overall concentration of dividing cells did not appear to be different from that of deeper regions of the lipophil zone (Fig. 7g,h). We also did not detect any definite pattern of EdU positive cells in the wound or elsewhere in the wounded animals. The average density of EdU positive cells in injured animals both near and away from the wound was slightly greater than in regions in the lipophil zone of intact animals (Fig. 7h), but no significant difference was found among these three groups with an ANOVA test (F = 1.668; p = 0.2295).

## Discussion

Our electron and light microscopy show that placozoan fiber cells are capable of motility and engage in phagocytosis. Fiber cells enwrap and engulf dying cells and incorporate cell debris in phagosomes. Dissociated fiber cells maintained in vitro have motile processes and, when grown on Matrigel, can migrate. They engulf heat-killed *E. coli* and *S. aureus* bacteria and incorporate them into intracellular compartments that are acidic, as shown by staining with lysotracker, and contain reactive oxygen species, as shown by staining with DHE. Fiber cells also participate in wound healing: Their elongated shapes and positions near the cut edge suggest that they contribute to closure of the wound. We show by in situ hybridization that fiber cells express genes homologous to transcription factor *foxC* and *pxmp2*, thus confirming that the cell cluster exhibiting high expression of these genes in RNAseq analyses^39^ was correctly identified as representing fiber cells. They also express genes encoding for proteins implicated in actin-myosin mediated motility, phagocytosis and innate immunity (^39^; present study).

Early literature about *Trichoplax* cell types asserted that fiber cells are contractile and responsible for generating rapid changes in body shape that are characteristic of the animal^14,30,37^. That dissociated fiber cells extend and retract processes (^30,38^, present study) supports the idea that they are contractile, but whether fiber cells generate changes in body shape remains to be determined. Instead, body shape changes may be produced by contraction and relaxation of dorsal and ventral epithelial cells^25,50^ and by forces generated by the beating cilia of VEC^17,19^.

Based on the presence of plug junctions between fiber cells and evidence of anastomoses between fiber cell in vitro it was earlier proposed that fiber cells are a syncytium^30^. Our SBF-SEM studies of fiber cells do not support this hypothesis; we did not observe anastomoses among the five fiber cells we reconstructed. Although the image resolution we used would not allow distinction between syncytial and plug junctions, the cells would appear anastomosed if connected in either way. The scarcity of plug junctions observed in TEM studies on Placozoa in the present study and previous studies^15,30^, together with the absence of evidence for anastomoses between fiber cells in situ casts doubt on the idea that fiber cells perform an integrative role^27,30^.

### Fiber cells and innate immunity

Innate immunity is the main defense mechanism in invertebrates. It provides clearing of cell debris, self/non-self recognition, and non-specific pathogen elimination^51^. The *Trichoplax* genome and transcriptome include homologs of many immunity related genes in Cnidaria and Bilateria^52–54^. However, cellular mechanisms of immunity had not been described in placozoans. We found that the fiber cell is the only cell type in *Trichoplax* that phagocytizes cell debris and bacteria.

Cell turnover in *Trichoplax* is fast and cell death occurs constitutively^55^ thereby creating a need to eliminate dead cells. Dying and dead cells display surface signals that target them for engulfment by professional phagocytes (e.g. macrophages) or amateur phagocytes (cells that engage in phagocytosis when activated by signals from injured cells)^56^. That fiber cells are distributed in a self-avoiding manner provides an optimal and efficient coverage of cells throughout the entire body; at the same time, the distance between fiber cell bodies allows their processes to reach out to the neighboring fiber cells. We observed fiber cell processes wrapped around cells that were intact but showed signs of being unhealthy, such as the presence of swollen ER and vacuoles. Enwrapment by fiber cell processes may be an initial step toward internalization. The wide flat processes of fiber cells may facilitate internalization by enclosing the targeted cells in their extended surface. Fiber cells with phagosomes containing cellular debris at varying degrees of degradation also were found.

That fiber cells phagocytose bacteria suggests that they also have a role in defense against pathogens. Likewise, the presence of large phagocytosed food particles, such as whole algal or yeast cells in fiber cell phagosomes^36^, could represent an immune/defensive response rather than a nutritive function. Fiber cells may encounter pathogens and food particles during sporadic breaches in the epithelium caused by the animals’ movements^50^. The ability of fiber cells to phagocytose bacteria may have provided the gate through which a rickettsial endosymbiont^57^ entered fiber cells during evolution, as was proposed for amoebae and their endosymbionts^58,59^ and for Cnidaria and their symbiotic algae^60^.

We found particles in the extracellular space that resembled the residual bodies of fiber cells as well as those in phagocytic cells in other animals^61–64^. It is possible that the extracellular particles represent residual bodies extruded from living or dead fiber cells. The presence of a membrane surrounding the extracellular residual bodies precludes the possibility that they were extruded by classical exocytosis as are, for example, the contents of phagosomes in *Dictyostelium*^65^. However, the archeocytes in a fresh water sponge extrude their residual bodies into membrane enclosed vacuoles via a process that resembles exocytosis^66^. It has been suggested that food remnants are expelled through breaches in the dorsal epithelium of *Trichoplax*^36^. However, we found evidence suggesting that residual bodies may be extruded through a gap between cells in the ventral epithelium.

Immune cells in Bilateria and Cnidaria recognize cell debris and extracellular pathogens with Toll-Like-Receptors (TLR) and scavenger receptors on their plasma membrane^67–69^. No gene for a TLR was found in the *Trichoplax* genome, although homologs of the extracellular and intracellular domains of TLR were identified^6,52^. The *Trichoplax* genome encodes a large repertoire of scavenger receptors^6,52^, and we showed that one of them, *cd36*, is expressed by fiber cells. Apart from cell debris, CD36 also binds *E. coli* and *S. aureus* membranes^70–72^, and thus could trigger internalization of these bacteria by fiber cells.

In situ hybridization showed that nearly all fiber cells express homologs of *foxC*, *cd36*, *elmo*, and *pxmp2*. FoxC is a transcription factor expressed in mesoderm-derived cells in Bilateria^41^ and in the somatic gonad in Cnidaria, a structure whose expression profile includes genes associated with bilaterian mesoderm and endoderm^40^. ElMo is a component of the signaling pathway that mediates cytoskeletal rearrangements for phagocytosis and cell motility in *C. elegans* and mammals^73–75^; it is expressed in amoeboid cells with presumed immune functions inhabiting mesohyl of the demosponge *Amphimedon queenslandica*^76^. Peroxisomes, which may be the pale organelles that cluster with mitochondria in fiber cells, are implicated in ROS generation and innate immunity in bilaterians^77^ and porifera^78^. The fiber cell cluster identified by RNAseq^39^ expressed multiple genes involved in cell motility and production of components of the extracellular matrix, such as collagen and fibronectin.

### Fiber cells and wound healing

We examined wound healing in *Trichoplax* that had been cut in half. Time lapse imaging showed that the wound healed almost completely within one hour, as reported^79^. Immunostaining with an antibody that outlined fiber cells revealed that fiber cells were aligned along the cut edge within five minutes after wounding. If fiber cells are contractile, as are activated fibroblasts that participate in wound healing in mammals^80^, they could help to close the wound by shortening. Extracellular matrix produced by fiber cells^39^ might help to seal the wound. Physical sealing of the wound apparently is rapid since no fluorescent dextran dissolved in ASW after wounding penetrated the wounded animals, in contrast to the rapid penetration of dextran into animals maintained in calcium-free seawater, conditions that open adherens junctions between epithelial cells^18^. Constriction of dorsal and ventral epithelial cells near the wound site also may contribute to the gradual reduction in the extent of the wound that precedes wound closure^50^.

Proliferating cells were abundant in intact *Trichoplax* and no significant change in proliferation was apparent in wounded animals, indicating that repair of edge tissue and regeneration in *Trichoplax* occurs primarily through rearrangement of existing cells (morphallaxis) rather than generation and differentiation of additional cells (epimorphosis)^81–84^.

### Comparison of placozoan fiber cells to macrophage-like cells in other animals

The behaviors we document in *Trichoplax* fiber cells—motility, phagocytosis of cell debris and pathogens, participation in wound healing—are characteristic of macrophage-like cells that reside in different tissues in vertebrates^85^ and invertebrates^62^. Mammalian macrophages can self-renew in homeostasis and in response to challenges such as injury or inflammation^86^.

Phagocytic mesenchymal cells characterized by possession of motile processes and capability for migration are present in some cnidarians. Anthozoa and Scyphozoa have mesenchymal phagocytic cells in their mesoglea, called amoebocytes that participate in wound healing and bacterial phagocytosis (reviewed by^87,88^) and transfer nutrients between cells^89,90^. Hydrozoans lack motile mesogleal amoebocytes, and their wound healing process relies entirely on epithelial cells^91–93^. Hydrozoa contain motile stem cells, called interstitial cells (I-cells), which are located in the ectoderm and endoderm and differentiate into neurons, gland cells, nematocysts or germ cells; their ultrastructure is typical for stem cells and they do not contain phagosomes^61,94,95^. Although non-hydrozoans lack I-cells, their epithelial cells undergo mitosis and during regeneration can generate other cell types^61,96^. Non-hydrozoan amoebocytes rarely undergo mitosis and there is scarce evidence that they self-renew or transdifferentiate^96^.

Ctenophora mesoglea contains amoeboid cells called stellate cells that phagocytose cell debris and bacteria^97,98^. A role for stellate cells in wound healing has been demonstrated in *Mnemiopsis leidyi*^98,99^. Wound healing does not require cell proliferation. Ctenophora are well known for their ability to regenerate body parts. This process requires proliferation of populations of stem cells most of which are located near the tentacles^99,100^.

The mesohyl in many sponges (Porifera) is populated by amoeboid archeocytes that phagocytose pathogens and cell debris, and participate in wound healing (reviewed by^101^). Archeocytes rapidly accumulate at sites of injury and transdifferentiate into pinacocytes, which then are involved in wound epithelization^101^. Amoeboid cells are sparse or absent in Calcarea and Homoscleromorpha poriferans. In these poriferan classes, wound healing occurs by spreading and fusion of epithelial cell sheets^101^.

Motile mesenchymal cells that participate in wound healing and innate immunity are present in all metazoan phyla, although not in all species within the phyla. Macrophages in vertebrates can self-renew and, in some cases, play the role of stem cells^86^ but whether cnidarian amoebocytes, ctenophore stellate cells and placozoan fiber cells are stem cells remains to be determined. In situ hybridization with probes for *piwi*, a gene that is expressed in stem cells of animals^102^, could help to resolve this question.

### Roles of fiber cells in *Trichoplax* homeostasis

As fiber cells are the only cells in *Trichoplax* that phagocytose other cells, they likely are responsible for removing apoptotic cells, which are reported to be prevalent throughout the body^55^. Being fragile animals, *Trichoplax* in their native habitat likely are prone to injury by predators or inanimate objects. The rapid sealing of wounds evident from the minimal entry of dextran into cut animals would prevent entry of pathogens. Ruptures to the epithelium that occur during binary fission^103^ are very limited in extent and probably also seal rapidly. However, large ruptures in the dorsal and/or ventral epithelium are repaired much more gradually^25,50^ leaving ample time for invasion of pathogens. Phagocytic uptake of bacteria by fiber cells would help to protect vulnerable host cells from these invaders. Although we found no evidence suggesting that epithelial cell types phagocytose bacteria, epithelial cells potentially could participate in protection against pathogens by producing anti-microbial peptides/toxins, as do epithelial cells in Hydra^104^ and some types of epithelial cells in Bilateria^105^. A more comprehensive analysis of the gene expression profiles of fiber cells could reveal the signaling pathways and effectors that mediate their homeostatic functions and provide more evidence to support or refute hypotheses about possible roles for fiber cells in intercellular communication or motility.

### Significance to understanding metazoan evolution

Multiple approaches have been used to characterize functions, morphology, and diversity of cell types in representatives of each metazoan phylum as well as in unicellular holozoans in order to gain insight into the evolutionary origins of Metazoa (reviewed by^53,106–112^). Four somatic cell types previously have been identified in representatives of all animal phyla: squamous/cuboidal/columnar epithelial cells that form a protective barrier, absorptive epithelial cells that take up nutrients, mucocytes that secrete mucus, and sensory cells that secrete peptides and/or small molecular transmitters^14,20,24,62,76,113–122^. Now that is apparent that placozoan fiber cell perform macrophage-like functions, we have evidence of a fifth ubiquitous cell type: the motile macrophage. More information about the gene regulatory pathways and expression profiles of cell types in non-bilaterian metazoan phyla is needed to establish whether cells that perform analogous functions are homologs. Nevertheless, the near ubiquity of these five cell types across metazoan phyla suggests that they probably were present in the ancestral metazoan^53,107,114,123,124^. This ancestral organism likely also possessed germ cells since almost all eukaryotes are capable of sexual reproduction^107^.

Metazoans are widely thought to be the descendants of a colony of cells similar to modern choanoflagellates^109,125^, although alternative scenarios also have been proposed^111,112,126^. The genomes of the ancestral cells likely contained the large set of genes shared between Metazoa and Holozoa, including some that are absent in modern choanoflagellates^110,112,127^. Many innovations were needed to transform a simple colony of ciliated cells, each able to feed and reproduce, into an obligate multicellular individual containing differentiated somatic cell types and germ cells arranged in a consistent pattern. Each of the four *epithelial* cell types that are thought to have been present in the common ancestor of Metazoa—barrier, absorbtive, sensory secretory, and mucus secretory cell—exhibits a distinct subset of cytoskeletal structures and organelles present in Choanoflagellates maintained under ordinary (non-confined) conditions^34^. The evolution of the different epithelial cell types is hypothesized to have occurred by “division of labor” of functions carried out by the ancestral Choanoflagellate-like cell^107,109,125,128^. The differentiation of the fifth cell type that is hypothesized to be ancestral in Metazoa, the amoeboid macrophage-like cell, required transformation of epithelial cells into mesenchymal cells. Epithelial-mesenchymal transitions (EMT) occur during development and regeneration in all metazoan phyla and homologs of some of the key proteins involved in this process (Par, Brachyury, Snail, Runx) are conserved^129–133^. Some of the proteins involved in orchestrating EMT (Brachyury, Snail, Runx transcription factors, cytoskeletal proteins involved in amoeboid motility) are expressed in Filasteria and Pluriformea, holozoans related to choanoflagellates and metazoans and known for their ability to transform from flagellated to amoeboid cell phenotypes^125,126,134^. This has led several authors to suggest that motile mesenchymal cells appeared early in metazoan evolution by coopting genes involved in temporal cell type transitions in their unicellular/colonial relatives^112,125–127^.

The ancestor of animals likely was a filter feeder, like Choanoflagellates and Porifera^29,107,109,125,135^. Evolving an animal that could feed by extracellular digestion required another cell type, an exocrine cell that secreted digestive enzymes. The invention of external digestion and predation are proposed to have initiated an “evolutionary arms race”^136^ that led to the diversification of animal body plans and lifestyles that occurred in Ctenophora, Cnidaria and most exuberantly in Bilateria. Although Placozoa evolved external digestion, they retained, or perhaps secondarily evolved, a uniquely simple body plan and lifestyle providing us the opportunity to study an animal that may resemble the ancestor of animals on earth today. Research on *Trichoplax* has elucidated strategies that this ancestor may have employed to move, locate and eat prey, maintain homeostasis and recover from injury, and protect itself from pathogens.

## Methods

### Animals

*Trichoplax adhaerens* (Schultze, 1883) of the Grell (1971) strain, a gift from Leo Buss (Yale University), were kept in Petri dishes with artificial seawater (ASW; Instant Ocean, Blacksburg, VA, USA) supplemented with 1% Micro Algae Grow (Florida Aqua Farms, Dade City, FL, USA) and red algae (*Rhodomonas salina*, Provasoli-Guillard National Center for Culture of Marine Plankton, East Boothbay, ME, USA), as described previously^137^. Water was partially changed once a week.

### Electron microscopy

Sample preparation (high pressure freezing, freeze substitution, and embedding) were performed as described elsewhere^15^. Transmission electron microscopy (TEM) was done on a JEOL-200-CX (Tokyo, Japan) microscope with an AMT camera mounted below the column at 120 kV.

Serial sectioning on an Automatic Tape-collecting Lathe Ultramicrotome (ATLUM) with subsequent scanning electron microscopy (SEM) in backscatter mode were performed as described previously^26^. For serial blockface scanning electron microscopy (SBF-SEM), the specimens were cut from epon and trimmed before mounting on an aluminum pin with conducting epoxy. SBF-SEM imaging was done with a Gatan 3View system mounted inside a Zeiss Sigma SEM at 1.2 kV. The block was sectioned in 100 nm Z-steps and micrographs taken at 10 nm in XY, with image sizes of 40 µm × 60 µm.

### Segmentation and rendering surface models

Images obtained by ATLUM/SEM and SBF-SEM were imported to EM3D, electron tomography software package (em3d.org). Cells were segmented from images and rendered using EM3D and customized programs^138–140^. Volumes of interest containing individual fiber, lipophil, epithelial, and gland cells were manually segmented throughout reconstructed volumes using EM3D. Because the cellular membranes were heavily stained and irregular, their volumes of interest (VOI) were defined by manually marking a closed path on the series of slices in which they were included. Typically, the VOIs were slightly larger than the structures that they enclosed to allow optimal surface models; however, the manual delineation of such refined VOIs is tedious and time-consuming. Here instead of such repetitive manual refinement of the VOIs and surface models, the automatic segmentation method^138^ was applied to each of the roughly segmented VOI to effectively produce its segmented structures as shown in Figs. 1 and 2 and Supplementary Movies 1–3.

### Cell dissociation

To dissociate the cells, a group of animals was rinsed in calcium and magnesium free ASW prepared as described previously^141^ and then incubated in 0.25% trypsin in calcium-free ASW for 2 h. Then the animals were transferred to normal ASW where they were triturated with a glass Pasteur pipette until the suspension was homogeneous.

### Recording of the behavior of fiber cells

Dissociated cells were placed in Lab-Tek II coverslip chambers (Thermo Fisher Scientific, Waltham, MA). Time-lapse images with 10 s interval were collected with a LSM 800 confocal microscope with a 63X 1.4 NA objective and fluorescence and DIC optics (Carl Zeiss Microscopy LLC, Thornwood, NY, USA). Fiber cell membranes were counterstained with fluorescein-concanavalin A (# FL-1001, Vector Laboratories, Burlingame, CA, USA). In order to observe fiber cell motility, the glass bottom of the chambers was covered with a thin layer of ECM Gel from Engelbreth-Holm-Swarm murine sarcoma (# E1270, Millipore, Burlington, MA, USA), a substrate that cells adhered to less strongly than to glass.

### Phagocytosis assay

This analysis was performed with dissociated cells, obtained as described above. The cell suspension was supplemented with fluorescent heat killed bacteria (0.1 mg/ml, by Life Technologies, Eugene, OR, USA), a vital dye, and Hoechst nuclear stain (# H-3570, Molecular Probes, Eugene, OR, USA). Bacteria conjugated with Alexa Fluor™ 594 (*E. coli*, # E23370, or *S. aureus*, # S23372) were added along with Lysotracker Green DND-26 (0.3 μM, # L7526, Invitrogen, Eugene, OR, USA). LysoTracker was used to visualize lysosomes and phagosomes with low pH content; it also labels lipophil cell granules^17^. Bacteria conjugated with Alexa Fluor™ 488 (*E. coli*, # E13231, or *S. aureus* # S23371) were added along with Dihydroethidium (10 μM, # D23107, Life Technologies, Eugene, OR, USA). Dihydroethidium was used to detect reactive oxygen species. Unlike other oxidants, superoxide anion oxidizes dihydroethidium to 2-hydroxy-ethidium which has an excitation peak at 405 nm^142,143^ and has an affinity for DNA. 2-hydroxy-ethidium generated in the interior of a cell labels DNA in the nucleus, DNA-containing organelles (e.g., mitochondria) and intracellular bacteria, if present. Here we used 2-hydroxy-ethidium staining to detect uptake of bacteria by fiber cells.

The suspension was transferred to Lab-Tek II coverslip chambers and left on a shaker in the dark for 45 min. Images were collected with a LSM 800 confocal microscope with a 63X 1.4 NA objective and fluorescence and DIC optics.

### Fluorescence in situ hybridization

In situ hybridization was performed with probes and reagents from Advanced Cell Diagnostics, Inc. (ACD, Hayward, CA, USA) using protocols developed to optimize staining in *Trichoplax*^24,144^. Sequences of *Trichoplax* orthologs of CD36, FoxC, and PXMP2 have been published previously^39,52^. ElMo ortholog was found using NCBI database. The candidate amino acid sequence was analyzed with the free online tool for classification of protein families (InterPro). The confirmed sequence demonstrated high similarity to respective orthologs in other animals, as shown by BLAST against non-redundant protein sequences of eukaryotes. Phylogenetic analysis was done in the NCBI database using pairwise alignment of the closest sequences in non-vertebrate animals (Supplementary Fig. S5).

The RNA sequences were retrieved from a *Trichoplax* transcriptome database^54,145^, access to which we have been granted by Adriano Senatore (University of Toronto). The regions encoding conservative domains of the proteins (see Supplementary File) were used to generate Z-probes, which were designed by ACD using the proprietary RNAscope Probe Design pipeline.

Animals or cells, dissociated as described above, were transferred onto cover slips with a drop of ASW with 0.97 M mannitol (in water) mixed 1:1. Samples were left on cover slips to adhere for about 2 h. Then the liquid was blotted, and the cover slips were plunged into prechilled tetrahydrofuran on dry ice and kept overnight (in some experiments, animals were cut in half with a scalpel prior freezing).

The cover slips with animals were then transferred to 3% acetic acid in methanol at − 20 °C for 30 min followed by a mixture of formalin and methanol (1:10), initially at − 20 °C and then at room temperature (RT) for 30 min. The samples were rinsed twice in methanol, dried for 5 min and then treated with Protease IV for 30 min.

The cover slips with dissociated cells were transferred directly to a mixture of formalin and methanol (1:10), initially at − 20 °C and then at RT for 30 min. The samples were then rinsed once with methanol, twice with ethanol followed by descending concentrations of ethanol in PBS (70% and 50%) and PBS. The cells were treated with Protease III diluted 1:15 in PBS for 15 min.

Hybridization was performed with RNAscope Fluorescent Multiplex Reagent Kit (# 320850) according to supplier’s instructions using the following RNAscope probes: Ta-*foxC* (# 810611-C2), Ta-*elmo* (# 830061), Ta-*cd36* (# 828391) and Ta-*pxmp2* (# 810621). The negative control included application of 3-Plex Negative Control Probe (# 320871), which gave no labelling. Samples were counterstained with 1:200 wheat germ agglutinin (WGA) conjugated to Alexa 647 (# W32466, Thermo Fisher Scientific, Waltham, MA) or CF405M (# 29028, Biotium, Hayward, CA, USA) and DAPI, mounted in ProLong™ Gold antifade reagent (# P36934, Invitrogen, Eugene, OR, USA), and examined in LSM 800 or LSM 880 confocal microscope.

Labelling with each probe was done at least twice; the results of independently repeated experiments were similar. In wholemounts, each preparation included about 10 animals. For each preparation with macerated cells several 51 × 51 μm^2^ fields were scanned. Cell counts (Supplementary Table S2) were then made for each field and the percent of expressing fiber cells, spherical/columnar epithelial cells, and lipophils was calculated. The cell was considered labeled if it possessed at least one fluorescent grain. Mean percent of expressing cells was then obtained by averaging the data from all fields, and standard deviation (StDev) and standard error of mean (SEM) were calculated.

### Immunostaining

*Trichoplax* were placed on coverslips with a drop of ASW and mannitol mixture and left until they flattened and adhered. The animals (except for a control group) were cut in half with a scalpel and frozen after the desired period of healing in prechilled tetrahydrofuran on dry ice. Samples were freeze substituted as described previously^15,146^ and incubated overnight in the primary rabbit antibody against a peptide (Ac-LDNPLRESRTSRYC-amide) from the predicted *Trichoplax adhaerens* classical cadherin sequence TaCDH^147^, custom made by New England Peptide (Gardner, MA, USA). This antibody, referred to here as anti-fiber cell (anti-FC), has been identified as a cell type marker that binds to fiber cell surfaces^15^. After rinsing, samples were incubated in a mixture of secondary Atto 488 goat anti-rabbit antibody (# 18772, Sigma-Aldrich, St Louis, MO, USA), Alexa 647-WGA, and Hoechst dye. Samples were mounted in ProLong™ Gold antifade reagent (Invitrogen, Eugene, OR, USA), and examined with a LSM 800 or LSM 880 confocal microscope.

### Analysis of fiber cell distribution

*Trichoplax* were labelled with anti-FC antibodies and imaged as described above. The images were then processed in Fiji software. Flat regions of body were cropped and optical sections encompassing fiber cell layer were projected into a flat image. The center of each fiber cell was manually labelled with a dot, and separate images having dots only were created. These images were uploaded to BioVoxxel Toolbox^49^ where the nearest neighbor distances were automatically measured, and distribution of the dots was evaluated with ‘2D Particle Distribution’ plugin. The manner of dot distribution was inferred based on the comparison (Welch's t-test) of the average and median nearest neighbor distances with a hypothetical nearest neighbor distance characterizing random distribution of dots. Five images obtained from five animals were evaluated.

### In vivo observation of wound healing

*Trichoplax* were transferred to a Warner RC-40LP chamber (#1.5 cover glass substrate; Warner Instruments, Hamden, CT, USA) containing 2 ml ASW with 1:1000 WGA conjugated to Alexa 647. The chamber was placed on the microscope (LSM 800 confocal) stage and the animal was viewed with 10X 0.30 NA objective. Merged DIC and fluorescence images (1024 × 1024 resolution) were collected continuously (no delay between frames) with an acquisition time of 3.7 s/frame. A short time series of the animal was captured. Image acquisition was stopped while the animal was cut with a scalpel and image acquisition was then resumed. The width of the wound was measured manually with Freehand Line tool in Fiji on the images at one-minute intervals.

### Dextran penetration during wound healing

Four groups of *Trichoplax* each consisting of 2–3 animals were transferred into Lab-Tek II coverslip chambers and left to adhere to the bottom. Wound permeability was probed with 70 kDa FITC conjugated dextran (# D1823, Life Technologies, Carlsbad, CA, USA), a dextran too large to readily diffuse through the intercellular junctions between *Trichoplax* epithelial cells^18^. The dextran was dialyzed against ASW and diluted in ASW at a final concentration of 0.25 mg/ml. Dextran was added to an intact (control) group of animals and to a group of animals that were wounded *after* dextran application by cutting them in halves with acupuncture needles. The other two groups of animals were wounded in the same manner prior to adding dextran at 30 s or 60 s.

The chambers were left for 50 min in the dark to allow dextran to penetrate into the wound and then the animals were examined on a LSM 510 confocal microscope (Carl Zeiss Microscopy, LLC). Fields of view included a randomly chosen part of the animal body and an outside space with brightly stained ASW.

This experiment was repeated thrice with each group yielding 3–6 images. Mean fluorescence of the scanned *Trichoplax* body and the outside space was then measured in Fiji software. The ratio of inside to outside mean fluorescence for each image was measured and averaged among the animals in each group. ANOVA was applied to evaluate the statistical differences between the four groups.

### Proliferative activity measurement

We used Click-iT™ EdU Cell Proliferation Kit with Alexa Fluor™ 555 dye (# C10338, Invitrogen, Eugene, OR, USA) to visualize dividing cells. Two groups of *Trichoplax* were placed on cover slips with a drop of ASW. Once the animals attached, EdU (Component A) was added to both groups at a final concentration of 0.1 mM. *Trichoplax* in one of the groups were then immediately cut in half with a scalpel, while *Trichoplax* in the other group (control) were left intact. Both groups were fixed 35 min after EdU application with a cocktail of paraformaldehyde (4%, EMS, Hatfield, PA, USA) and glutaraldehyde (0.25%; EMS, Hatfield, PA, USA) in buffered ASW (NaCl 400 mM; MgCl_2_ 5 mM; CaCl_2_ 2 mM; sucrose 300 mM; HEPES 30 mM; pH 7.4) for 2 h. The samples were processed according to the manufacturer’s protocol, stained with a mixture of fluorescein-WGA (# FL-1021-10, Vector Laboratories, Burlingame, CA, USA) and Hoechst dye, and mounted in Vectashield (Vector Laboratories, Burlingame, CA, USA).

The samples were examined on a Zeiss LSM 800 confocal microscope. Two randomly chosen non-overlapping regions were scanned in each animal in the control group. Cut animals were scanned in a wounded area and in an area at least 100 μm away from the wound. Images were then analyzed using Fiji software. EdU + nuclei were counted in 100 × 100 μm^2^ area within the lipophil zone, which begins in approximately 80 μm from the edge of the body. The number of EdU + nuclei was then averaged for each group; in the group of wounded animals, mean value was calculated separately for wounded area and away from the wound. ANOVA was used to analyze the difference between these three groups.

## Supplementary Information

Supplementary Information 1.

Supplementary Movie 1.

Supplementary Movie 2.

Supplementary Movie 3.

Supplementary Movie 4.

Supplementary Movie 5.

## Data Availability

All data generated during the research is shown in the manuscript.
