# Supplementary material for: Placozoan fiber cells: mediators of innate immunity and participants in wound healing
Source: Sci Rep. 2021 Dec 2;11:23343. doi: 10.1038/s41598-021-02735-9 (PMC8639732; doi:10.1038/s41598-021-02735-9)
Supplement: Supplementary file 1 — Supplementary Information 1. [file 41598_2021_2735_MOESM1_ESM.docx]

**Supplementary Information**

**Placozoan fiber cells: Mediators of innate immunity and participants in wound healing**

Tatiana D. Mayorova^1,*^, Katherine Hammar^2^, Jae H. Jung^1^, Maria A. Aronova^3^, Guofeng Zhang^3^, Christine A. Winters^1^, Thomas S. Reese^1^, Carolyn L. Smith^4^

1 – Laboratory of Neurobiology, National Institute of Neurological Disorders and Stroke, National Institutes of Health, 49 Convent Drive, Bethesda, MD 20892, USA

2 – Central Microscopy Facility, Marine Biological Laboratory, 7 MBL Street, Woods Hole, MA 02543, USA

3 – Laboratory of Cellular Imaging and Macromolecular Biophysics, National Institute of Biomedical Imaging and Bioengineering, National Institutes of Health, 9000 Rockville Pike, Bethesda, MD 20892, USA

4 – Light Imaging Facility, National Institute of Neurological Disorders and Stroke, National Institutes of Health, 35 Convent Drive, Bethesda, MD 20892, USA

* – Author for correspondence (tatiana.mayorova@nih.gov; [mayorova@wsbs-msu.ru](mailto:mayorova@wsbs-msu.ru))


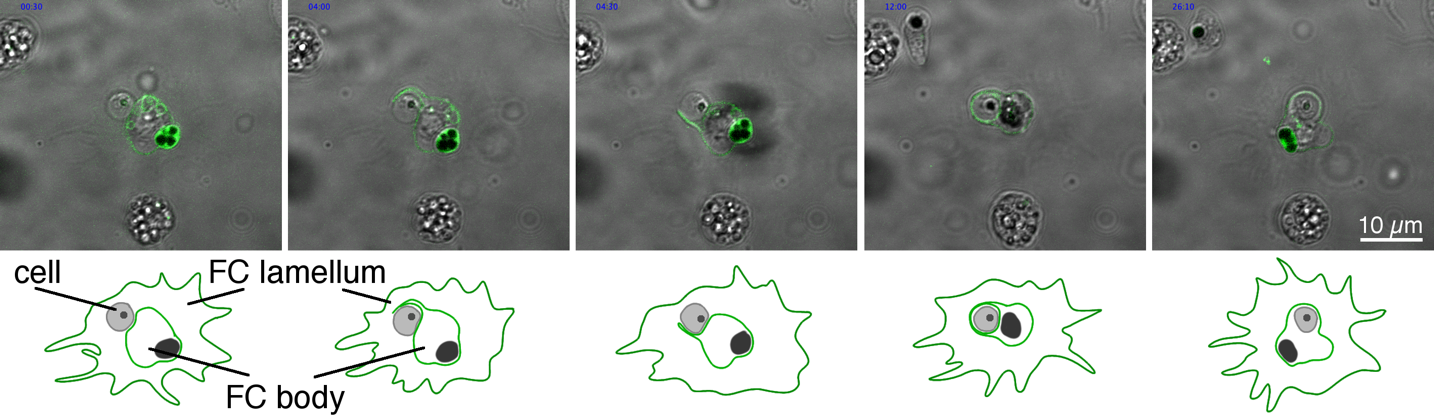


**Fig. S1**.  Series of still shots from Supplementary Movie S4 accompanied with drawings showing outlines of the fiber cell body and lamellum (green) and the small cell (gray) that the fiber cell engulfed.  Time is shown in min:sec.  The fiber cell membrane was stained with fluorescein-concanavalin A.  Processes of the fiber cell probed the small cell and then fused to surround it.  Eventually, the small cell appeared to be in the interior of the fiber cell because it was enclosed within the fiber cell membrane.

**
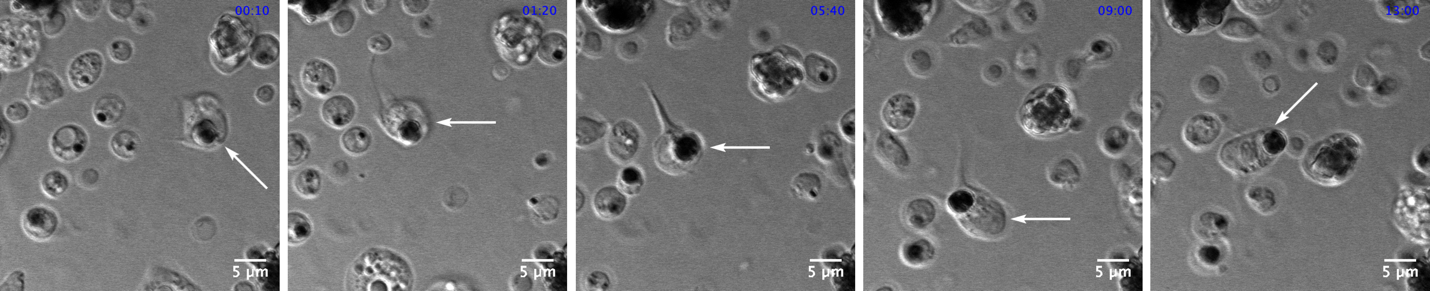
**

**Fig. S2**. *Trichoplax* dissociated cells seeded on ECM Gel from Engelbreth-Holm-Swarm murine sarcoma (Matrigel), to which cells adhere less strongly than to glass. Images are still shots of the same area taken at different time intervals (upper right corner; min:sec). Arrow indicates a fiber cell migrating and projecting processes in different directions. Other cells are motile due to possession of a beating cilium.

**
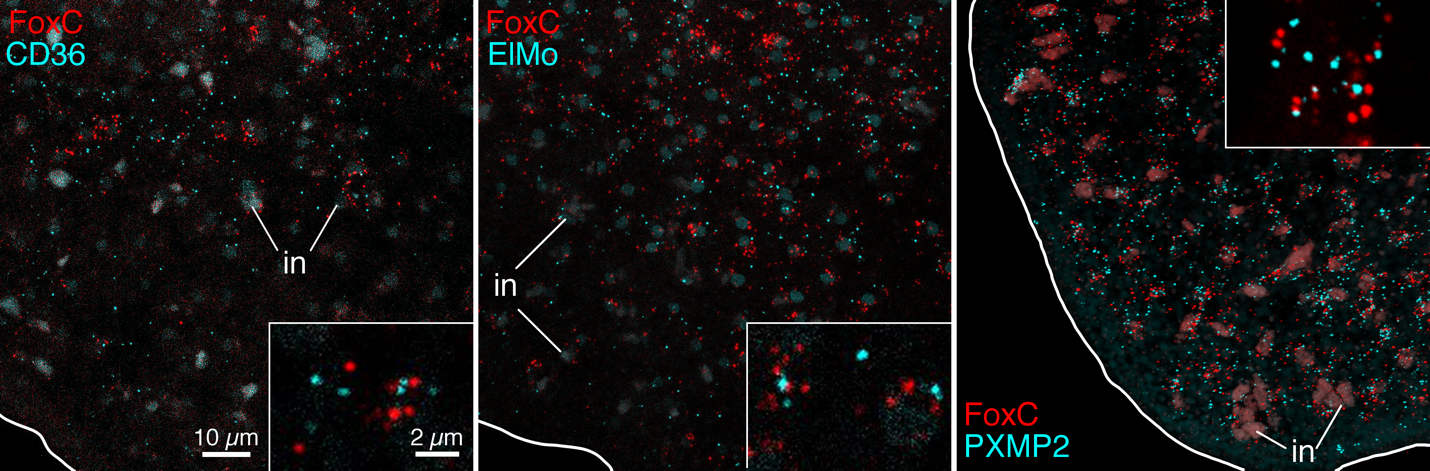
**

**Fig. S3**. In situ hybridization in wholemounts with probes for the transcription factor *foxc* and genes implicated in phagocytosis. Panels show *foxC* in red along with different genes: *cd36*, *elmo*, and *pxmp2* in cyan. Images are maximum projections of optical sections through the entire thickness of the animal. Note patchy pattern of expression of these genes and the proximity of grain clusters to autofluorescent fiber cell inclusions (in). White line marks the edge of the animal body. Insets are single optical sections showing co-expression (close apposition of grains labeled with two different probes implying single cell localization) of *foxC* (red) and the respective phagocytic gene probe (cyan).

**
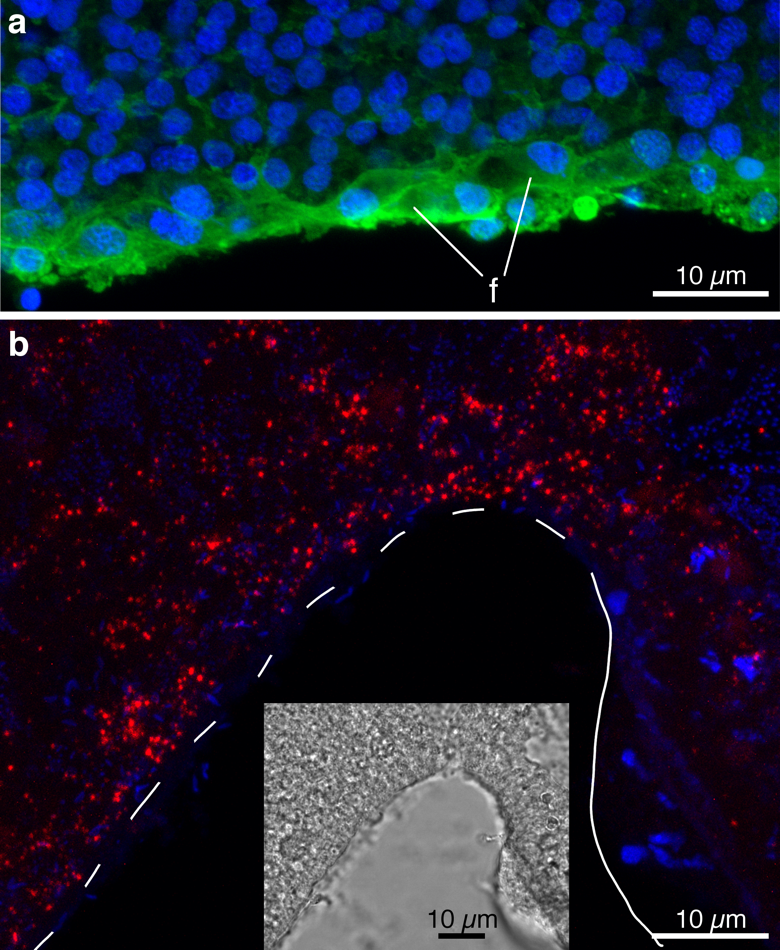
Fig. S4**. Fiber cell involvement in the wound healing. **(a)** anti-FC (green) immunolabelling of fiber cells (f) accumulated at the cut edge 5 minutes after surgery. Hoechst nuclear stain, blue. **(b)** *foxC* expression (red) along the cut edge confirms the accumulation of fiber cells in this area. The line marks intact edge where solid, and cut edge where dashed. Mucocytes labelled with WGA (blue) are seen in the intact area. Inset shows the sample in transmitted light.

| 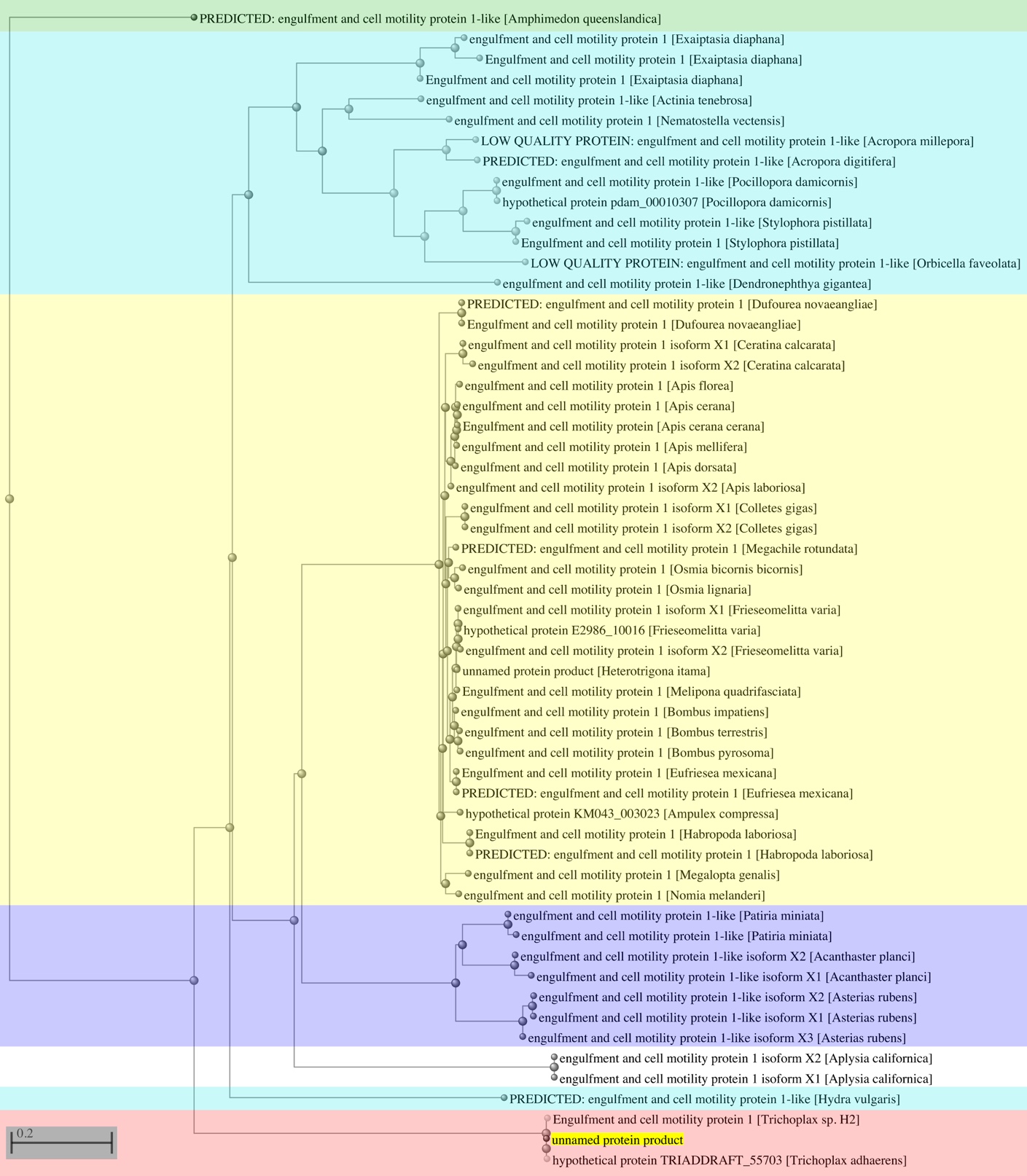 |
| --- |

**Fig. S5**. Phylogenetic tree built from an alignment of the sequences with similarity to *Trichoplax* ElMo ortholog (unnamed protein product) using fast minimum evolution method (max seq difference = 0.7, distance = Grishin protein). Color coding: Porifera – green; Cnidaria – cyan; Ecdysozoa – yellow; Deuterostomia – lavender; Lophotrochozoa – white; Placozoa – pink.

**Movie S1.** Movie of rotating 3D surface rendering of a fiber cell (green) and adjacent cells: two VECs (yellow) and a Type 2 gland cell (red). Constructed from serial thin sections obtained by ATLUM/SEM.

**Movie S2.** Movie of rotating 3D surface rendering of two fiber cells (different shades of green) and four lipophil cells (different shades of magenta). Constructed from serial thin sections obtained by ATLUM/SEM.

**Movie S3.** Movie of rotating 3D surface rendering of five fiber cells located in the lipophil zone. Constructed from serial thin sections obtained by SBF-SEM.

**Movie S4**. Engulfment of a small round cell by a fiber cell recorded in a culture of dissociated cells. Time is shown in min:sec. The fiber cell membrane was stained with fluorescein-concanavalin A; the fluorescence was detected at the focal plane of the fiber cell body. Processes of the fiber cell probed the small cell and then fused to surround the cell. After ~30 min, the small cell appeared to be in the interior of the fiber cell because it was completely enclosed within the fiber cell membrane and moved similarly to other inclusions within the fiber cell.

**Movie S5**. Wound healing in a *Trichoplax* cut in half. Time is shown in min:sec format. The intact edge of the animal is evident due to staining with WGA-647 (red) and the presence of birefringent crystals in crystal cells, which flash as they change orientation with respect to the polarized transmitted light. The animal moved so the stage was adjusted to keep it in the field of view. Though the wound opened periodically, it gradually shrank and finally the lips of the wound fused, leaving a small notch marking the site of the wound.

**Table S1**. Measurements of the nearest neighbor distance of fiber cells and statistical comparison with random distribution.

|  | Image 1 | Image 2 | Image 3 | Image 4 | Image 5 |
| --- | --- | --- | --- | --- | --- |
| Frame size, width x length, µm | 137.55  x  129.56 | \| 114.23  x  127.03 \| \| --- \| | \| 88.60  x  89.06 \| \| --- \| | \| 155.41  x  135.17 \| \| --- \| | \| 247.54  x  333.09 \| \| --- \| |
| Fiber cell number | 110 | 104 | 45 | 146 | 278 |
| Theoretical random nearest neighbor dis-tance, µm ± St. Dev. | 6.36±0.98 | 5.91±0.93 | 6.41±0.77 | 6.00±1.00 | 8.47±2.18 |
| Measured average nearest neighbor dis-tance, µm ± St. Dev. | 8.97±1.90 | 7.44±2.05 | 9.32±2.20 | 7.99±2.40 | 10.34±2.69 |
| Measured median nearest neighbor dis-tance, µm ± St. Dev. | 8.92±1.90 | 7.25±2.06 | 9.76±2.24 | 7.81±2.41 | 10.13±2.70 |
| t (Welch's t-test) | 12.50 | 6.07 | 9.48 | 8.37 | 7.99 |

**Table** **S2**. The total number of cells in RNAscope experiments counted and evaluated for the presence of expression.

|  | Fields of view | Fiber cells | Epithelial cells | Lipophils |
| --- | --- | --- | --- | --- |
| FoxC | 33 | 66 | 736 | 34 |
| CD36 | 15 | 48 | 393 | 62 |
| ElMo | 21 | 35 | 852 | 20 |
| PXMP2 | 33 | 66 | 736 | 34 |

Region of *Trichoplax adhaerens foxC* homolog sequence used to generate Ta-*foxC* probe

gtatggatatgaatgcctataatgccaatggtcaaaagaatttaacacctaccaacgatttaattacgccacatcctggcactacacattccgtgtatgctcctgataataattacagtagttatgaagtttatagagctggccatccttatccgacgattgctccaccacatctttcgttacagatgatgccatcacatgctagcggcgatcaatatcctaatgttgctgcggtacctgttgccgttgctgctaaggaccattcagtttctttaatgtcaagtcatactggagcgcaattttattcaactcctggtgctgttagccaacgctattcaacagcaatgactaaccctgctactggaactgctattgctgcacctagcacgactaaagataatgcagttaaaccagcttattcctatattgcattaattgctatggctattaagagcaagcatgatcgcaaaattaccttaaatggtatttatcaatttatcatggaccattttccttactacagacaaaataagcaaggatggcaaaatagtattaggcataatttaagcctaaatgaatgctttgttaaggtaccacgtgaagaaggtgaaaagcgaccgaaaaagggcaactactggacattgcatccagatagtgaaaatatgtttgaaaatggaagttttctacggcgacgtaaacgtttcaagagaaattcttcaagtagcagcactagcaatactaacaatagcagtagcgaactacagcagcaatcatatcatcatcagcatcatcaacaccaccatcagcaacaacagcaagacgtcaataataaattatcaaatgattgtttagcaagcgatcataaaggtaaagtatcggataagaatcatcatgatagtaatagtaaattattagatgttcaaccatcttcttcatcatcttctacacgaagtttgcctaaagctgaaataggtgaaacaacagcagtcaataatgatgttaatcagtcatcgtctccactaccatcaacactccacgaccaatcgcatcacaccctaacatcattaattaatcaaaaagatcatccaagtaaatgtaaaattgagaacgacggccatgatcaatcgagccatgagaatagcagtataccgcatacttcggttgccgatatcaattcaacaggtagtgcttcagtaggaactttgggatcatcaaattcagccagatcaagccccaatactgctagtggtaaaaataacatgcaaagaattgttaattatccctttgttgattaccccgtcaataaatggcctggtggaactggcaatcattattatacagctggcactttatcctatccaagcggtattgctgtcgtatcacctggacaactttgtactgctccagctgctgtttccagccaatttctcgaagctcataatacaaatccagttgtaaccaagcatgatgaatcgcaacaacaacagcaatcacaacaacagcagcaacaacaacaacatcttataccatcatattggcaatcaccagctgttgctacaggaagtactaatcagacacaaaatcattctaatagttatcgtgatatctacgatatgcaacgttacagtggtggcctttgcacatctgctcctcaaatttattcgagaatgggtccaatg

Region of *Trichoplax adhaerens cd36* homolog sequence used to generate Ta-*cd36* probe

tcatggcatgtacctgcggttgtgttgccatcactacgctggttttgggtgttttgctagcgattggatgtggtgttggtattccgctactaacgacgttcttgagcggagtagttgatcagagggtagaattattaccgaataccactggttataataattggctgcatcctggcggaccaatttatatgcaattttatattttcaatattgtgaatccattagaaattgagcaaggtcaacgaccggctgttgaacaaattggcccatttacttatagggaacatcgtattaaaactgatgttgtcttctacaccaatggcactgcatcttacaatgagaagaagatttttatttttctaccagatcgatcaattgatagcgataattttacgtttacaactatcaacgtaccattattaacaacgttggaaattatgaggaaagataatatatcagaaaatcttaagaaactctttttcaatttcatttcaaacctgcataacgagggattgtttataaagcggtcagttagacagatgatatggggctatgatgacgatatttttgactttttacgtgatataagtgcactgttgcctaacccagtcaaaattccgaaagtgtttggattgcaacaaaatgcctcaagtgctggaatatttgatgtttacactggtaaagatgatccgaaaaatttaggaaagattgcgcgttggaatggggaaagtcatttgcattggtggaaagacaaatatgctaatatgatcaatggaactgatgccgtacaatttcatccacatttgactcggaaggaaatgctatatttattcaataacgatgtatgcagatccttatactctatttaccactctgatgttaaactgaaaggcattaatttgtatcgttatactattcccggtaaagttttcttgaattcttcagctaatccagacaatgccggtttctgtgaaccgtgcctggcttctggactattaagtttatcctcgtgcaaacaaggggctcctgtagctatttcatcaccccatttcctttatggcgatcctcaattagtacataatgtaacaggtctacatccaaacttggaagagcatgcgacgtggattgatattgatccgattacaggctttaccatgcatgcccggaaacggttacaaattaatgctgtcgtctcgcctgataattcgcttaagggaatgaaaaatgtaaaatatgtagtaatgccgattgtttacctgaacgagagtgctgagattgataagaagcacgtcgacctgtataaccagcagcttactataccatttctaatcttccatattttggagtatgcggtgcctgcatttagtggaattcttatcctagtatcagcggtggttctatttcgtattaagcgacatggccgaattctgcacgtgcaacgatcagattatgagcccatagaata

Region of *Trichoplax adhaerens elmo* homolog sequence used to generate Ta-*elmo* probe

agtaaactggatcagctacgaaaaacagttcgcgaagcaggtgtctatgctgttgctggcgctgatgaagagattgaaatgaataacgaaacggatttcaagatcttaggcagtatgaatccaagagatcccacacttgacttaaacgatgaaccatctggtctcttggctttagataatatgattttcttctccaataaacaaaacgacaattttaggaagtttattttggaaaattgtggatgtaatgatagtcaagcttgtccattcatgaaatcaagtattgcactgacaaagttattgtgcaatctactaaaaattggagatgcatcattttccttaaatgagaatgattcctacatcgaagtatttttcgattccgatacggttttcgaggaattattttgcgtctgtattcaaatttggagcaagacatggaaagaaatgcatgctacttctgaagattttaacaaggttttaaatatcgttcaagagcaaattacaagaagtttg

Region of *Trichoplax adhaerens pxmp2* homolog sequence used to generate Ta-*pxmp2* probe

aatgtcggagaaaggcaaacaaaatatattgcatcggttgttagaccgatatttctatcttcttcaacactatccattggcaactaagtcaattacaagtgcgattcttgctggcttaggtgattttatatcacaaaaactagctcaaggtggtcaaggaaccatagtatggagaaacattggagcttacgccttttttaacttaatcgtgaccggaccgctctcgcatttctactatcagtggctggagaaactagttccatcgaaagtcccatttgctcctgctgtgcgagtcctagtggatcgtttgatatttgcaccaccgttcctcttcttagtcttctacttggtagcactctttcagggacattcatctgaagtggccatggataggattcgtaaatcctattggatatctttaaaaatgaattggcgtgtatggactgtattccaatatattatcgtcaattacgttccaacacattaccgtgtcttatgcggcaaccttgttggattgggctggaacatttacttatcatctaagcaaacctcct
